# Protective Effects of Bile Acids Against Hepatic Lipid Accumulation in Hybrid Grouper Fed a High-Lipid Diet

**DOI:** 10.3389/fnut.2022.813249

**Published:** 2022-01-25

**Authors:** Jia Xu, Xiaoyue Li, Xinzhou Yao, Shiwei Xie, Shuyan Chi, Shuang Zhang, Junming Cao, Beiping Tan

**Affiliations:** ^1^Laboratory of Aquatic Animal Nutrition and Feed, Fisheries College, Guangdong Ocean University, Zhanjiang, China; ^2^Aquatic Animals Precision Nutrition and High Efficiency Feed Engineering Research Center of Guangdong Province, Zhanjiang, China; ^3^Key Laboratory of Aquatic, Livestock and Poultry Feed Science and Technology in South China, Ministry of Agriculture, Zhanjiang, China

**Keywords:** high-fat diet, taurocholic acid, growth performance, lipid metabolism, fish

## Abstract

Bile acids (BAs) usually display growth-promoting and lipid-lowering properties when supplemented to the diet. The effects of a high-lipid diet (HD) and BAs supplementation on growth performance and lipid deposition of hybrid grouper (*Epinephelus fuscoguttatus*♀ × *E. lanceolatus*♂) was evaluated in this study. Compared to the control diet (CD), the HD did not significantly affect the fish growth performance, but it promoted lipid deposition, as revealed by a significantly higher crude lipid content of the whole body, muscle, and liver. Among the HD supplemented with taurocholic acid (BD) groups, and compared to the HD, fish fed dietary supplementation of BAs at 900 mg kg^−1^ exhibited the best growth performance and lowest hepatic lipid deposition. In most BD groups, the content of total cholesterol, low-density lipoprotein cholesterol, and triglycerides in serum, as well as the content of total cholesterol in the liver, were decreased, whereas the content of high-density lipoprotein cholesterol in serum was increased. In addition, the most strongly influenced pathways between the control, HD, and B3D groups were fatty acid biosynthesis, insulin signaling pathway, and AMPK signaling pathway. The improvement of lipid metabolism induced by the supplementation of BAs may be attributed to decreased expression of lipogenesis genes and proteins (enzymes), and increased lipolysis. In conclusion, dietary supplementation of BAs at 900 mg kg^−1^ promoted growth performance and reduced lipid accumulation, whereas BAs supplementation improved the hepatic lipid metabolism by enhancing hepatic lipolysis, inhibiting lipogenesis, and regulating associated transcriptional factors in hybrid grouper.

## Introduction

The hybrid grouper (*Epinephelus fuscoguttatus*♀ × *E. lanceolatus*♂) is a popular marine fish in Asia that has great potential in the aquaculture industry due to its rapid growth and popularity with consumers (1). Dietary lipids are considered to be among the most critical nutritional factors that affect fish growth (2). High-lipid diets (HD) for hybrid grouper are favored by the aquaculture industry because they reduce the use of costly protein as an energy source (3). However, excessive dietary lipids can cause lipid metabolism disorders, exacerbate triglyceride (TG) accumulation, increase the prevalence of non-alcoholic fatty liver disease (NAFLD), impair the liver function, and inhibit the growth performance of fish (4–6).

Bile acids (BAs) are synthesized from cholesterol, produced in the hepatocytes, stored in the gallbladder, and secreted into the intestine to facilitate the digestion of lipids and the absorption of triglycerides (TG), cholesterol, and lipid-soluble vitamins (7, 8). Previous studies showed that the addition of BAs improved the growth performance, including the weight gain (WG) and special growth rate (SGR), of largemouth bass (*Micropterus salmoides*) fed an HD (9) or a high-starch diet (10), of yellow croaker (*Larimichthys crocea*) fed an HD (11), of grass carp (*Ctenopharyngodon idella*) fed an HD (12), of tilapia (*Oreochromis niloticus*) fed a plant protein-based diet (13), of turbot (*Scophthalmus maximus*) fed a high plant protein diet (14), However, previous studies have not focused on the effects of dietary BAs on growth performance of hybrid grouper.

In addition, BAs play an important role in lipid metabolism (15, 16), while the anti-NAFLD effects have been well-documented by numerous studies in which BAs consumption reduced the weight of liver, serum and hepatic levels of total cholesterol (T-CHO) and TG, and improved morphological conditions of the liver in mice (17–19), and human subjects (18). Meanwhile, it was reported that BAs could reduce hepatic lipid accumulation in largemouth bass (9) and regulate hepatic lipid homeostasis in the tiger puffer (*Takifugu rubripes*) (5). However, previous studies have not investigated the effects of dietary BAs on lipid accumulation and metabolism of hybrid grouper.

Although studies have suggested that dietary BAs reduced lipid deposition in the liver of large yellow croaker, which could be attributed to the increased expression of lipid oxidation and decreased expression of lipid synthesis genes (11), it remains unknown whether this regulation pathway also exists in our focus fish, hybrid grouper. In this study, we evaluated the effects of HD and BAs supplementation on growth performance and lipid deposition of hybrid grouper. To gain a more complete picture, we explored the potential mechanisms by which dietary BAs regulated the lipid metabolism and decreased lipid accumulation in the liver. Understanding the functions of BAs may contribute to the development of management strategies for alleviating the negative impacts of a high-fat diet on growth performance and hepatic lipid accumulation in hybrid grouper.

## Materials and Methods

### Animals and Diet Preparation

This study was carried out following the recommendations for the Care and Use of Laboratory Animals in China, Animal Ethical and Welfare Committee of China Experimental Animal Society. The protocol was approved by the Animal Ethical and Welfare Committee of Guangdong Ocean University (Guangdong, China), processing ID: GDOU-AEWC- 20180063. The acclimation, feeding and rearing conditions of fish, and diet preparation are described in detail in the Supplementary Material.

### Feeding Experiment and Sample Collection

The control diet (CD, 8.27% lipid) and HD diet (15.32%, added soybean oil to the CD diet) were formulated according to the previous findings that 7–13% is an optimal dietary lipid level for grouper (≥15% lipid content causes fat accumulation in the liver) (4). As a 900/475 mg kg^−1^ supplemental level of BAs in a high fat/starch diet of largemouth bass significantly improved the lipid metabolism (9, 10), five BAs diets (BD) were prepared by adding taurocholic acid sodium (TCA, CAS: 345909-26-4, product code: T4009, purchased from Sigma Aldrich) levels at 300 (B1D), 600 (B2D), 900 (B3D), 1200 (B4D), and 1500 (B5D) mg kg^−1^ to the HD diet. The measured contents of TCA in diets (in mg kg^−1^) were as follows: CD = not detected, HD = 130.00, B1D = 393.30, B2D = 659.90, B3D = 888.10, B4D = 1,197.90, and B5D = 1,502.10. TCA was chosen as the focus BA on the basis of its highest proportion in this fish (not published) and other fish species (20). Detailed ingredients of CD, HD, and BD are provided in Table 1. The hybrid grouper specimens (*n* = 840; body weight = 7.8 ± 0.01 g) were randomly distributed into 28 plastic tanks (30 fish/tank, 500 L). These tanks were randomly assigned to seven groups (CD, HD, B1D, B2D, B3D, B4D, and B5D), ensuring four replicates per group (7 × 4 = 28). Full details are provided in the Supplementary Material.

**Table 1:** Composition and concentration of nutrients in diets^a^.

**Ingredients (%)**	**Test diets**						
	**CD**	**HD**	**B1D**	**B2D**	**B3D**	**B4D**	**B5D**
Fish meal	50.00	50.00	50.00	50.00	50.00	50.00	50.00
Vital wheat gluten	11.50	11.50	11.50	11.50	11.50	11.50	11.50
Wheat flour	15.00	15.00	14.97	14.94	14.91	14.88	14.85
Cottonseed protein	7.32	7.32	7.32	7.32	7.32	7.32	7.32
Corn gluten meal	2.00	2.00	2.00	2.00	2.00	2.00	2.00
Fish oil	2.00	2.00	2.00	2.00	2.00	2.00	2.00
Soybean oil	1.50	7.50	7.50	7.50	7.50	7.50	7.50
Soybean lecithin	2.00	2.00	2.00	2.00	2.00	2.00	2.00
Calcium monophosphate	1.00	1.00	1.00	1.00	1.00	1.00	1.00
Vitamin C	0.03	0.03	0.03	0.03	0.03	0.03	0.03
Choline chloride	0.50	0.50	0.50	0.50	0.50	0.50	0.50
Vitamin premix^b^	0.50	0.50	0.50	0.50	0.50	0.50	0.50
Mineral premix^c^	0.50	0.50	0.50	0.50	0.50	0.50	0.50
Antioxidant	0.05	0.05	0.05	0.05	0.05	0.05	0.05
Attractant	0.10	0.10	0.10	0.10	0.10	0.10	0.10
Cellulose microcrystalline	6.00	0.00	0.00	0.00	0.00	0.00	0.00
Taurocholic acid sodium^d^	0.00	0.00	0.03	0.06	0.09	0.12	0.15
**Proximate composition (% air dry matter)**							
Crude protein	47.48	47.53	47.62	47.51	47.42	47.45	47.64
Crude lipid	8.27	14.94	15.32	15.66	15.56	15.60	15.46
Crude ash	11.50	11.21	11.50	11.29	11.37	11.29	11.36
Moisture	8.94	10.01	9.55	9.71	7.97	11.16	9.78

a *Seven diets: CD (control), HD (high-lipid), B1D (taurocholic acid sodium additional level at 300 mg kg^−1^), B2D (600), B3D (900), B4D (1,200), and B5D (1,500)*.

b *Vitamin mixture (g/kg mixture): vitamin B1, 17.00 g; vitamin B2, 16.67 g; vitamin B6, 33.33 g; vitamin B12, 0.07 g; vitamin K, 3.33 g; vitamin E, 66.00 g; retinyl acetate, 6.67 g; vitamin D, 33.33 g, nicotinic acid, 67.33 g; D-calcium pantothenate, 40.67 g; biotin, 16.67 g; folic acid, 4.17 g; inositol, 102.04 g; cellulose, 592.72 g. All ingredients were diluted with corn starch to 1 kg*.

c *Mineral mixture (g/kg mixture): CaCO_3_, 350 g; NaH_2_PO_4_·H_2_O, 200 g; KH_2_PO_4_, 200 g; NaCl, 12 g; MgSO_4_·7H_2_O, 10g; FeSO_4_·7H_2_O, 2 g; MnSO_4_·7H_2_O, 2 g; AlCl_3_·6H_2_O, 1 g; CuCl_2_·2H_2_O, 1 g; KF, 1 g; NaMoO_4_·2H_2_O, 0.5 g; NaSeO_3_, 0.4 g; CoCl_2_·6H_2_O, 0.1 g; KI, 0.1 g; zeolite powder, 219.9 g. (Obtained from Zhanjiang Yuehai Feed Co. Ltd., Guangdong, China)*.

d *The measure value of taurocholic acid sodium: CD (no detected), HD (130.00 mg kg^−1^), B1D (393.30), B2D (659.90), B3D (888.10), B4D (1,197.90), and B5D (1,502.10)*.

### Growth Performance Analyses

The growth parameters were calculated: survival rate (SR), weight gain rate (WGR), specific growth rate (SGR), feed conversion ratio (FCR), feed intake (FI), viscerasomatic index (VSI), hepatosomatic index (HSI) and condition factor (CF). Full details for all these analyses are provided in the Supplementary Material.

### Diets and Body Composition

In brief, crude protein (*N* × 6.25) was determined following the Kjeldahl method after acid digestion using a Kjeltec system (Kjeltec 2300 Analyzer, Foss Tecator, Sweden); crude fat was evaluated by the ether extraction method using Soxtec System HT (Soxtec System HT6, Tecator, Sweden); moisture was determined by oven drying at 105°C until constant weight; crude ash was measured using a muffle furnace at 550°C until constant weight.

### Liver Staining Analyses

Samples were flash-frozen in liquid nitrogen, and the frozen tissues were sectioned (9 μm thickness), immersed in 1% oil red O working solution for 10 min, counterstained with hematoxylin, and then rinsed under running tap water for 30 min. Photomicrographs were captured with a light microscope under 200 × magnification. Integrated optical density (IOD) of the oil-red O stained areas was analyzed with Image-Pro Plus 6.0 software (Media Cybernetics, Silver Spring, MD, USA).

### Enzyme Activities and Biochemical Assays

The contents of TG (A110-1-1), T-CHO (A111-1-1), total protein (TP, A045-4) low-density lipoprotein cholesterol (LDL, A113-1-1), high-density lipoprotein cholesterol (HDL, A112-1-1), non-esterified fatty acid (NEFA, A042-2-1), and the activity of lipase (LPS, A054-2-1) were measured using commercial kits (Nanjing Jian Cheng Bioengineering Institute, Nanjing, China). The activities of adipose triglyceride lipase (ATGL, ml036372-2), carnitine palmitoyltransferase 1 (CPT1, ml09800-2), acetyl-CoA carboxylase (ACC, m1022714-2), and fatty acid synthase (FAS, ml036370-2) were determined through enzyme-linked immunosorbent assay (ELISA) kits (Shanghai Enzyme-linked Biotechnology Co., Ltd., Shanghai, China) following the manufacturer's instructions.

### The qPCR Analyses

The following genes (Supplementary Table S1) were selected: lipogenesis [6-phosphogluconate dehydrogenase (*6pgd*), *acc, fas*, 6-phosphate dehydrogenase (*g6pd*), and malic enzyme (*me*)]; lipolysis [*atgl, cpt1*, acyl CoA diacylglycerol acyltransferase 2 (*dgat*), diacylglycerol kinase alpha (*dgka*), hepatic lipase (*hl*), and hormone-sensitive lipase (*hsl*)]; fatty acid uptake [acyl-CoA binding protein (*acbp*), fatty acid-binding protein (*fabp*)]; transcriptional factors [liver X receptor alpha (*lxr*), peroxisome proliferator-activated receptor alpha (*ppara*), peroxisome proliferator-activated receptor gamma (*pparr*), and sterol responsive element binding protein 1 (*srebp1*)]; BAs receptors (*fxr* and *tgr5*); pro-inflammatory factors [interleukin 1β (*il1*β) and tumor necrosis factor-alpha (*tnf*α)]; anti-inflammatory factors (*il10*) and chemokine [chemokine ligand 8 (*cxcl8*)]. The details of total RNA extraction, cDNA preparation, and qPCR assays are provided in the Supplementary Material.

### Western Blot Analyses

The total protein isolation, denaturing, sodium dodecyl sulfate-polyacrylamide gel electrophoresis, transferring, blocking, incubation, visualization, and quantification assay were based on our published methods (21). The following antibodies were used: antibodies against FXR (1:500, bs-12867R, Bioss), TGR5 (1:500, NBP2-23669SS, Novus), SREBP1 (1:800, ab28481, Abcam), PPARA (1:1000, 66836-1-Ig, Proteintech), phosphor-PPARA (P-PPARA, S12) (1:800, ab3484, Abcam), and GAPDH (1:1,000, 2118S, Cell Signaling Technology).

### Determining the Levels of TCA in Diets

The levels of TCA in diets were determined using high-performance liquid chromatography-tandem mass spectrometry (HPLC-MS/MS) on the Ultimate3000-API 3200Q TRAP (USA). The HPLC-MS/MS system consisted of an SRD-3600 Solvent Rack with an analytical 6-channel vacuum degasser, a DGP-3600A pump, WPS-3000TSL analytical autosampler, and a tcc-3200 column compartment. Chromatographic separations were performed on an MSLab C18 column (150 × 4.6 mm, 5 μm). The mobile phase A was 5% acetonitrile (ACN) in water, and the organic mobile phase B was 25% isopropanol and 5% water in ACN. The solvent was delivered to the column at a flow rate of 1 ml min^−1^ as follows: 0–1 min from A–B (90:10) to A–B (90:10); 1–7 min from A–B (90:10) to A–B (5:95); 7–8 min from A–B (5:95) to A–B (5:95); 8–10 min from A–B (5:95) to A–B (90:10); 9–10 min from A–B (90:10) to A–B (90:10). The data was processed using Analyst software version 1.5.1 (Applied Biosystems). More details are described in the Supplementary Material.

### The Proteomic Analyses

The peptides were separated using a Shimadzu LC-20AB HPLC Pump system (Shimadzu, Kyoto, Japan) coupled with a high-pH RP column. The eluted peptides underwent nanoelectrospray ionization before being analyzed by the MS/MS (Orbitrap Fusion Lumos mass spectrometer; Thermo Fisher Scientific, San Jose, CA, USA) coupled with the nanoHPLC system. The raw data files were searched against the Uniprot *Perciformes*.fasta (299081sequences, release 2020_04) using the SEQUEST algorithm. The MS proteomics data have been deposited to the ProteomeXchange Consortium (http://proteomecentral.proteomexchange.org) via the iProX partner repository (22) with the dataset identifier PXD027928. Full details are provided in the Supplementary Material.

### Statistical Analysis

Results were presented as means ± standard deviation (SD) and data were tested for normality and homogeneity of variance using the Shapiro-Wilk and Levene's tests, respectively. The data were evaluated by one-way ANOVA and further analyzed by Duncan's multiple range tests. The analyses were performed with SPSS 23.0 (IBM, Armonk, NY, USA). Values of *p* < 0.05 were considered significant.

## Results

### Dietary BAs Improved the Growth Performance of Fish Fed an HD

**Figure 1:**
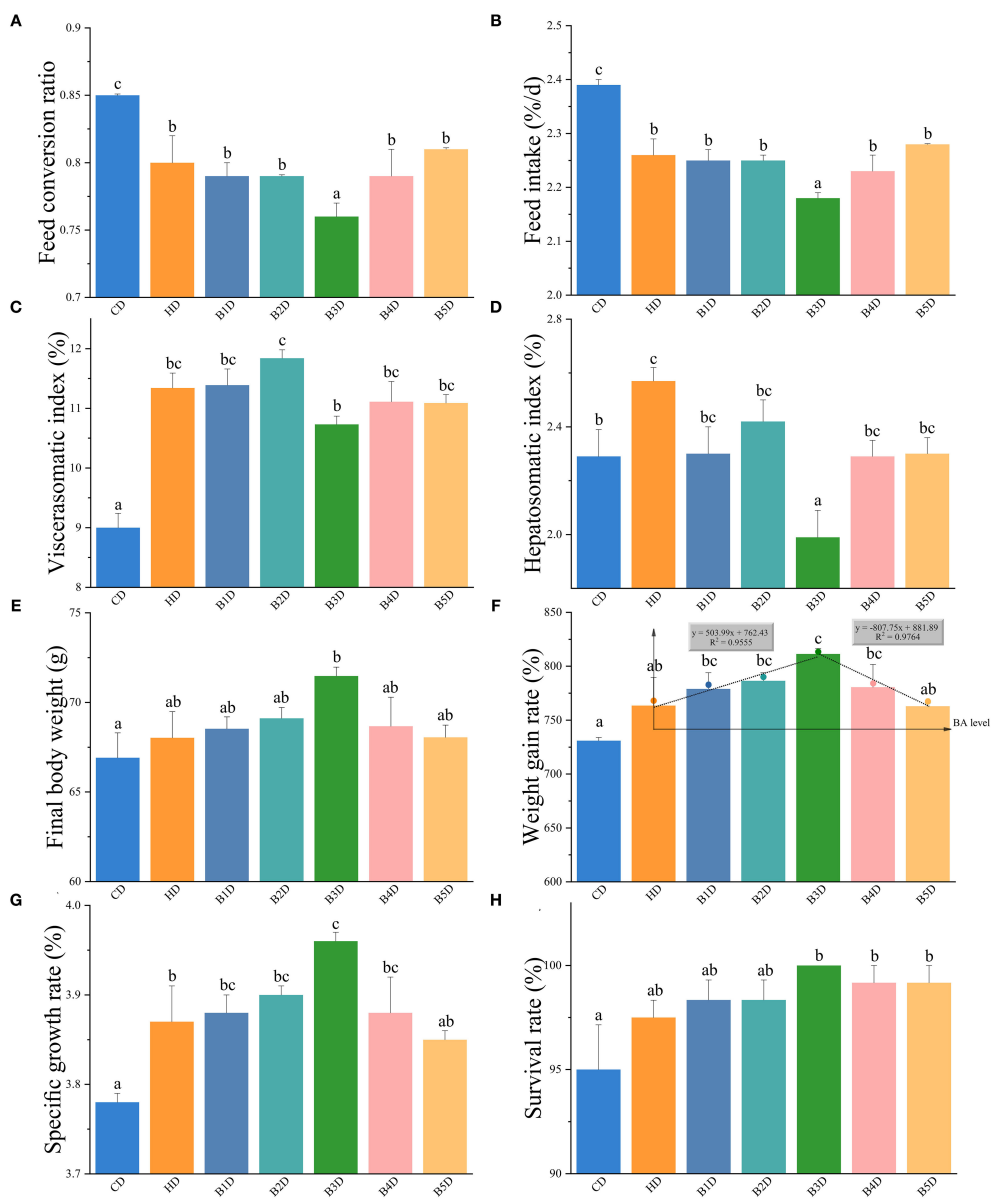
The growth performance of hybrid groupers from HD and BD groups. **(A–H)** the feed conversion ratio (FCR), feed intake (FI), viscerasomatic index (VSI), hepatosomatic index (HSI), final body weight (BW^F^), the weight gain rate (WGR), specific growth rate (SGR), and survival rate (SR) of hybrid grouper fed seven diets for 8 weeks (*n* = 4): CD (control), HD (high-lipid), B1D (taurocholic acid sodium additional level at 300 mg kg^−1^), B2D (600), B3D (900), B4D (1,200), and B5D (1,500), respectively. **(F)** broken line regression equation (*y* = 503.99*x* + 762.43, *R*^2^ = 0.9555; *y* = −807.75*x* + 881.89, *R*^2^ = 0.9764) results indicate that the optimal dietary BAs level in a HD diet is 900 mg kg^−1^. Values are presented as means with plus error bars (SD, standard deviation), where significant (*p* < 0.05) differences between groups are indicated by different letters.

The hybrid groupers were fed CD, HD, and BD (B1D, B2D, B3D, B4D, and B5D) for 8 weeks (unless if stated otherwise, all pairwise comparisons are HD vs. CD and BD vs. HD). In the HD group, the FCR and FI were significantly decreased, and the VSI and HSI were significantly increased (all *p* < 0.05, Figures 1A–D). After supplementation with BAs to the HD, the final body weight (BW^F^) and WGR in most BD groups (more than three groups) were increased, and the HSI values in all BD groups were decreased (Figures 1E,F). These parameters indicated that dietary BAs improved the fish growth performance. In addition, the WGR and SGR of the B3D group were the highest among the five BD groups, whereas the FCR, FI, and HSI were the lowest (Figures 1G,H). So, when the exogenous dietary BAs level was at 900 mg kg^−1^, fish exhibited the best growth performance, with a higher growth rate compared to other levels.

### High Dietary Lipids Impaired, While BAs Improved the Fish Lipid Deposition

Furthermore, HD consumption promoted lipid deposition, as revealed by a significantly higher crude lipid content of the whole body, muscle, and liver (all *p* < 0.05, Table 2). After the dietary supplementation of BAs, the crude lipid content of muscle and liver in most BD groups was decreased. In the BD group, the lowest content of crude lipid of the whole body, muscle, and liver was observed in the B1D, B4D, and B3D groups, respectively. These results implied that BAs intervention obviously reduced the lipid deposition induced by the HD in varying degrees and in different tissues.

**Table 2:** Average proximate composition of the whole-body, muscle, and liver in diet groups.

**Proximate composition (% of wet matter)**							
	**CD**	**HD**	**B1D**	**B2D**	**B3D**	**B4D**	**B5D**
**Whole-body**
Moisture	71.89 ± 0.31^a^	69.76 ± 0.14^b^	70.00 ± 0.18^b^	69.60 ± 0.11^b^	69.86 ± 0.33^b^	69.84 ± 0.42^b^	70.14 ± 0.54^b^
Crude protein	17.26 ± 0.24	17.24 ± 0.12	16.87 ± 0.16	16.75 ± 0.23	16.87 ± 0.28	16.82 ± 0.20	16.95 ± 0.24
Crude lipid	5.82 ± 0.11^a^	8.24 ± 0.12^c^	7.81 ± 0.07^b^	8.12 ± 0.02^bc^	8.38 ± 0.15^c^	8.33 ± 0.11^c^	8.74 ± 0.03^d^
Crude ash	4.05 ± 0.04^a^	4.04 ± 0.00^ab^	4.10 ± 0.02^a^	4.06 ± 0.01^a^	4.24 ± 0.07^b^	4.04 ± 0.05^a^	4.07 ± 0.07^a^
**Muscle**
Moisture	77.95 ± 0.07	77.31 ± 0.13	77.25 ± 0.15	77.88 ± 0.35	78.41 ± 0.13	77.95 ± 0.39	78.12 ± 0.97
Crude protein	20.52 ± 0.09^c^	20.05 ± 0.13^bc^	20.10 ± 0.13^bc^	19.75 ± 0.41^bc^	19.02 ± 0.22^ab^	19.86 ± 0.32^bc^	18.36 ± 0.76^a^
Crude lipid	0.82 ± 0.03^a^	1.66 ± 0.10^d^	1.71 ± 0.04^d^	1.61 ± 0.02^cd^	1.61 ± 0.01^cd^	1.30 ± 0.02^b^	1.52 ± 0.04^c^
Crude ash	1.34 ± 0.02^b^	1.36 ± 0.02^b^	1.48 ± 0.03^c^	1.26 ± 0.05^ab^	1.21 ± 0.01^a^	1.24 ± 0.01^a^	1.26 ± 0.04^ab^
**Liver**
Moisture	65.20 ± 1.21	66.07 ± 1.00	65.6 ± 0.18	64.09 ± 0.34	64.00 ± 0.87	64.75 ± 0.90	65.44 ± 1.06
Crude lipid	5.19 ± 0.16^a^	8.59 ± 0.48^de^	8.39 ± 0.51^cd^	7.44 ± 0.25^bc^	7.23 ± 0.41^b^	7.56 ± 0.30^bcd^	9.53 ± 0.11^e^

*Seven groups: CD (control), HD (high-lipid), B1D (taurocholic acid sodium additional level at 300 mg kg^−1^), B2D (600), B3D (900), B4D (1,200), and B5D (1,500). Values (n = 4) are presented as means with plus error bars (standard deviation), where significant (p < 0.05) differences between groups are indicated by different letters*.

**Figure 2:**
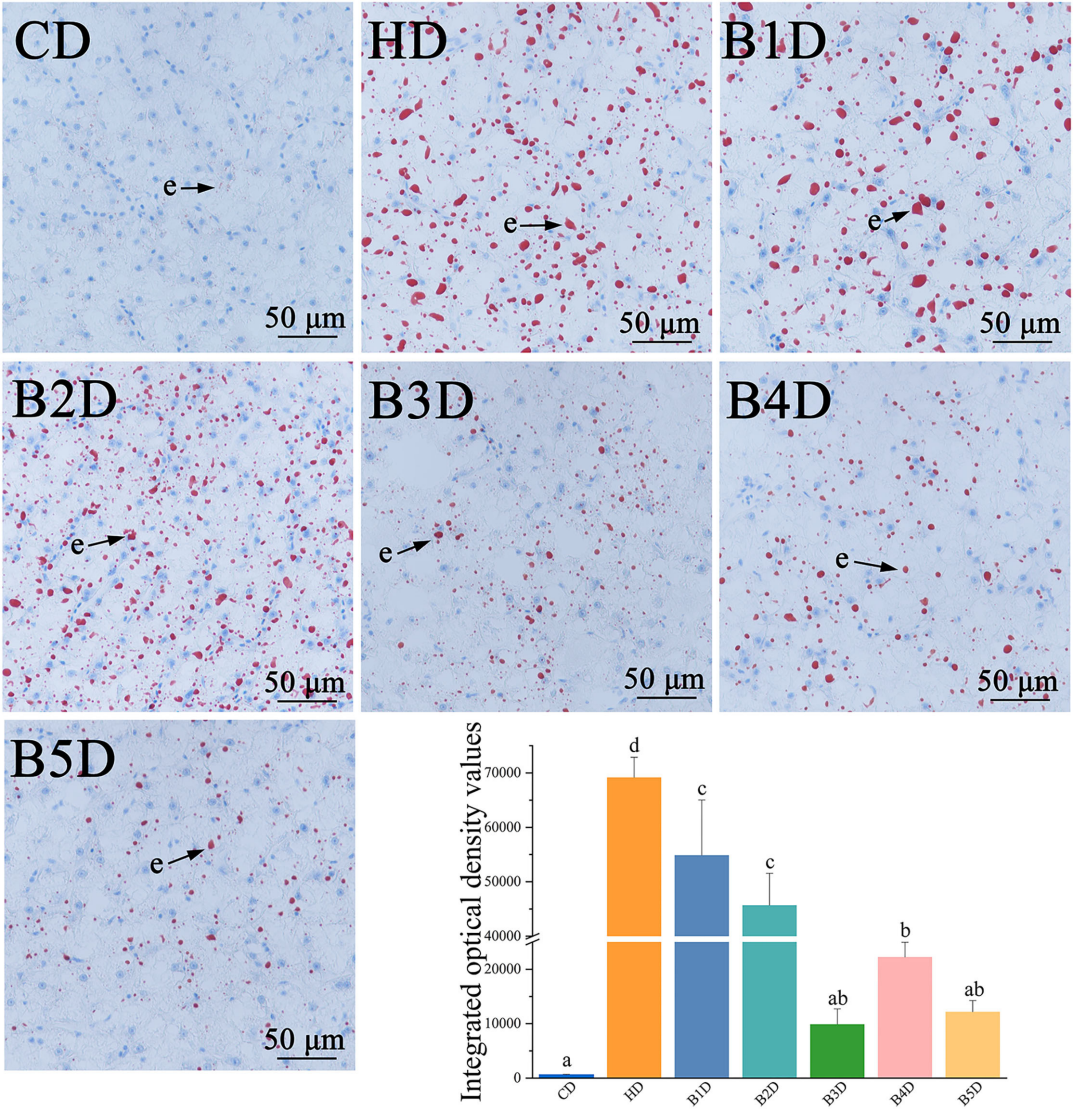
Photomicrographs of representative oil red O-stained histological liver sections of fish from HD and BD groups. e: lipid droplets are stained red and analyzed by integrated optical density (*n* = 4), and the nuclei are stained blue in oil-red O-stained sections. Values are presented as means with SD, where significant (*p* < 0.05) differences between groups are indicated by different letters.

Lipid accumulation in hepatocytes was elevated in the HD group, as reflected in the larger number of red-stained hepatocytes (lipids stain red by oil-red O), and significantly higher IOD values (Figure 2). The lipid accumulation in all BD groups was significantly decreased, as reflected in the small number of red-stained hepatocytes and lower IOD values (all *p* < 0.05), especially in the B3D and B5D groups. Overall, these results indicated that HD supplementation with 900 mg kg^−1^ BAs decreased hepatic lipid accumulation.

### High Dietary Lipids Impaired, While BAs Improved the Hepatic Lipid Metabolism

**Figure 3:**
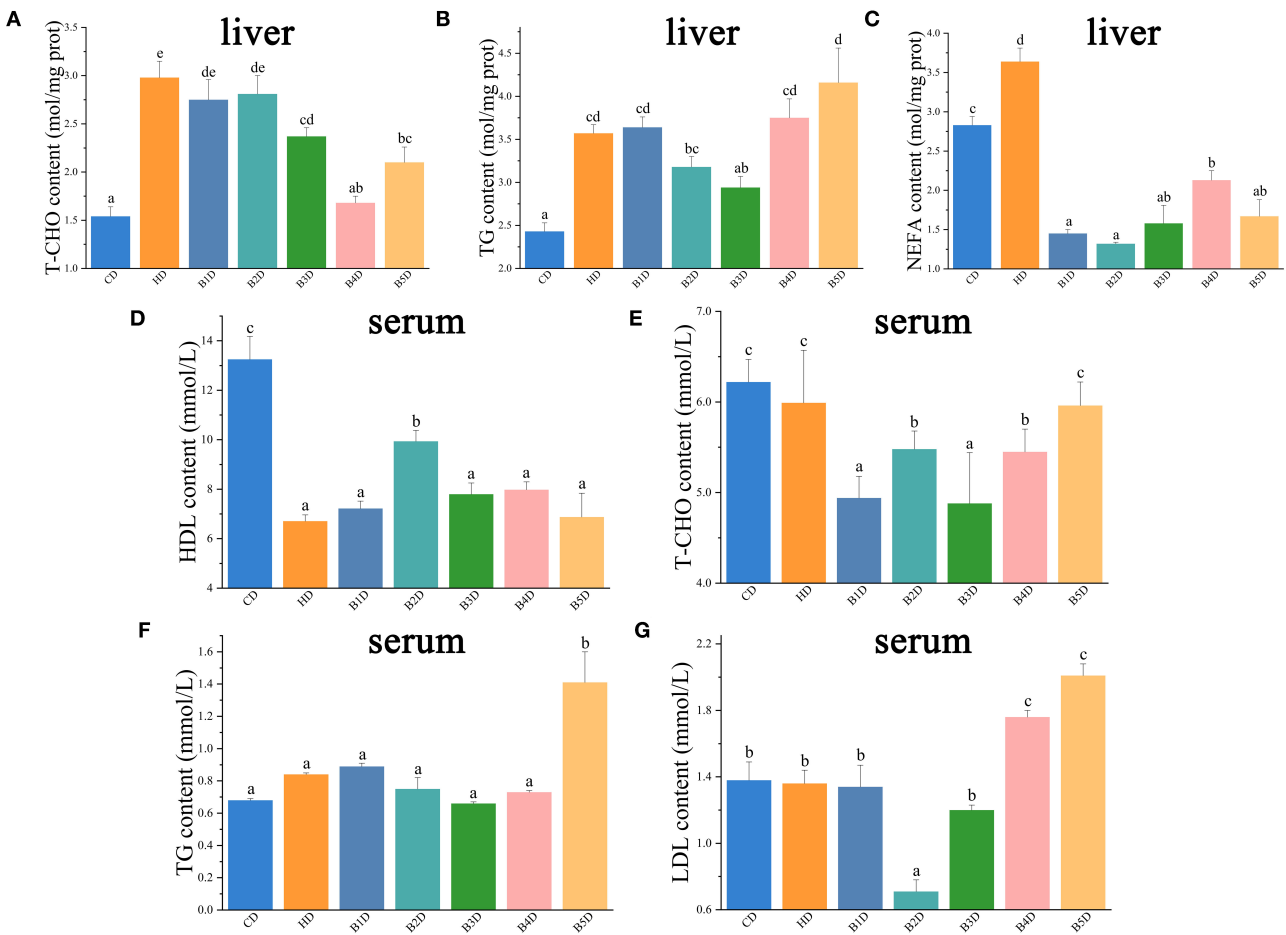
Biochemical indicators in liver and plasma of hybrid groupers from HD and BD groups. **(A**–**C)** the contents of T-CHO (total cholesterol), TG (triglycerides), and NEFA (non-esterified fatty acid) in the liver (*n* = 6). **(D**–**G)** the contents of HDL (high-density lipoprotein cholesterol), T-CHO, TG and LDL (low-density lipoprotein cholesterol) in serum (*n* = 6). Values are presented as means with SD, where significant (*p* < 0.05) differences between groups are indicated by different letters.

In the HD group, the content of T-CHO, TG, and NEFA in the liver were significantly increased, whereas the content of HDL in serum was significantly decreased (all *p* < 0.05, Figures 3A–D). In most of the BD groups, the content of T-CHO, LDL, and TG in serum, as well as the content of T-CHO and NEFA in the liver, were decreased, whereas the content of HDL in serum was increased (Figures 3E–G). These results indicated that the HD diet impaired the lipid metabolism in liver, and these adverse effects were apparently reversed with the addition of BAs.

### High Dietary Lipids and BAs Induced Changes of Fatty Acid Biosynthesis in the Proteomic Profile of Liver

**Figure 4:**
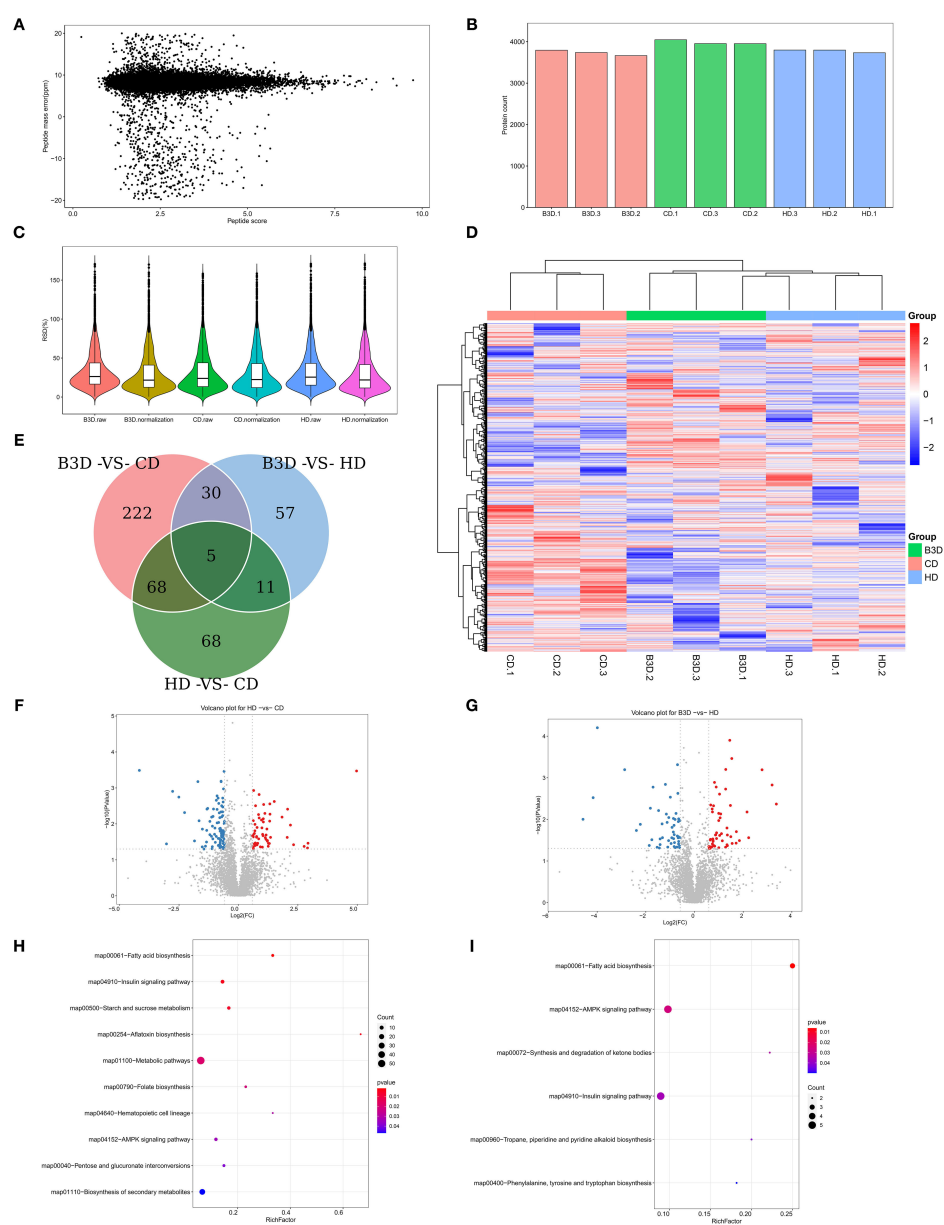
The basal proteomic profile analyses of the liver in CD, HD, and B3D groups. **(A)** The mass error of all detected peptides (*n* = 3). The *X*-axis represents the score of the peptide segment (the higher the better); the *Y*-axis represents the mass error (the smaller the better). **(B)** The number of identified proteins in all samples. **(C)** The comparison of CV before and after the normalization of identified proteins. CV (coefficient of variance) is the ratio of standard deviation to the mean of the abundance of proteins. After normalization, the CV decreased significantly, and lower CV means better overall repeatability of the sample. **(D)** The heatmap with Euclidean distance to perform hierarchical clustering of the abundance of proteins and samples simultaneously (*n* = 3). **(E)** the number of overlapping DEPs in Venn. Proteins with the fold change above 1.5 and *P*-value below 0.05 (using the Benjamini-Hochberg multiple testing correction test) were considered to be significantly differentially expressed proteins (DEPs). **(F,G)** The volcano plot of DEPs in the HD vs. CD group and B3D vs. HD group comparisons. The *X*-axis represents the fold change (log^2^ value) of DEPs, and *Y*-axis represents the *p*-value (–log_10_ value) of the fold change of DEPs. The gray dots represent proteins with no significant difference, the red dots represent the upregulated, and the blue dots represent the downregulated proteins. **(H,I)** the bubble diagram of DEPs in the GO enrichment analysis of the HD vs. CD group and B3D vs. HD group comparisons. The *X*-axis represents the Rich Factor, and *Y*-axis represents the name of a metabolic pathway in the GO enrichment. The Rich Factor is the ratio of a to b. a: the amount of DEPs in one pathway; b: the amount of all proteins in this pathway.

**Figure 5:**
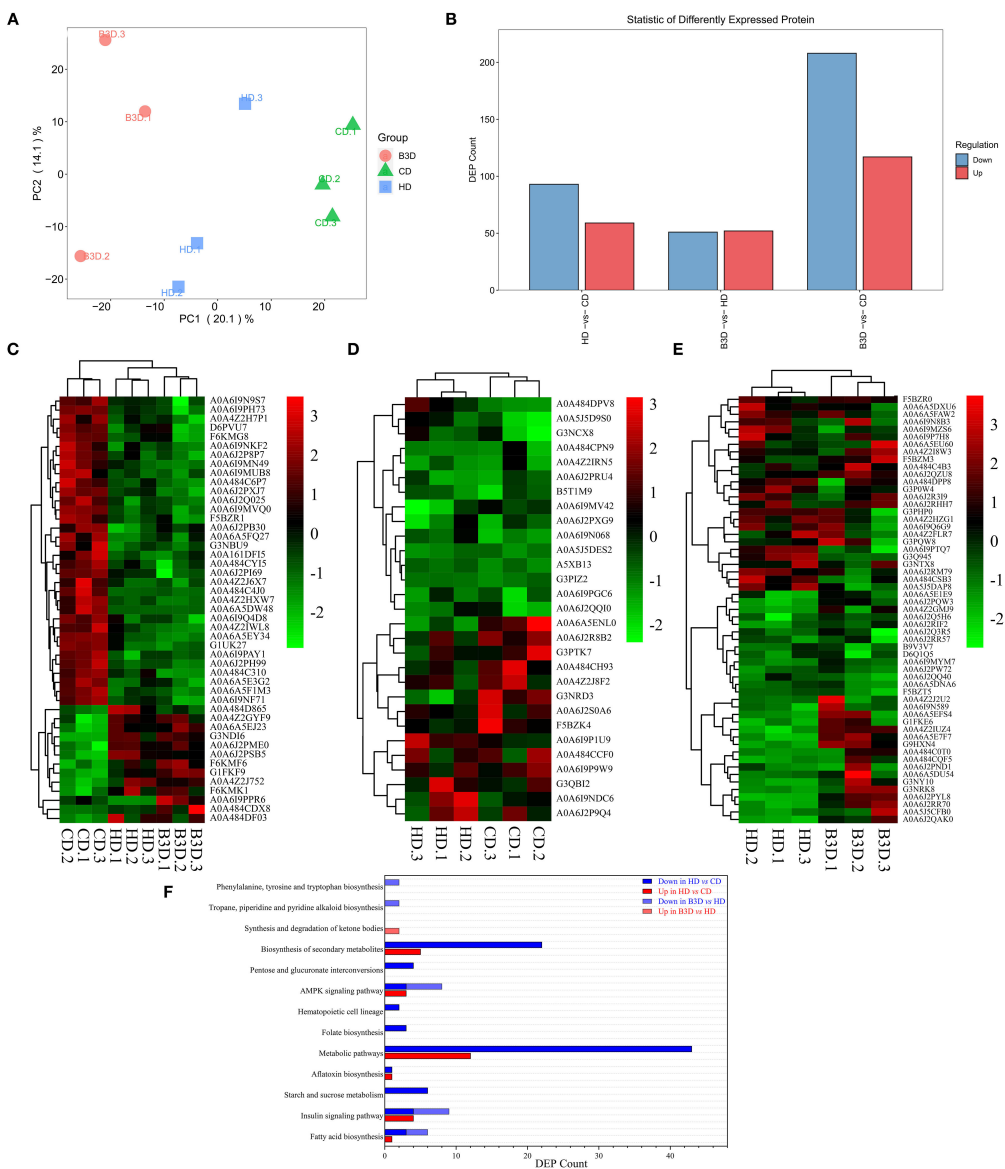
Further analyses of proteomic profiles of liver samples in CD, HD, and B3D groups. **(A)** the largest two weight-scores of principal components (PC) in the protein composition: PC1 explained 20.1% of the total variability of the data, and PC2 explained 14.1% (*n* = 3). **(B)** the amount of down or upregulated DEPs in the HD vs. CD, B3D vs. HD, and B3D vs. CD group comparisons. Proteins with a fold change larger than 1.5 and a *P*-value below 0.05 (using the Benjamini-Hochberg multiple testing correction test) were designated as DEPs. **(C)** The heatmap of DEPs in the HD group (compared to the CD group) and B3D group (compared to the HD group). **(D)** The heatmap of DEPs in the HD group (compared to the CD group). **(E)** the heatmap of DEPs in the B3D group (compared to the HD group). **(F)** the amount of down or upregulated DEPs in the KEGG enrichment analysis of HD vs. CD and B3D vs. HD group comparisons.

Based on the growth performance and lipid deposition of fish, proteomic analyses were performed on liver samples of the CD, HD, and B3D groups to ensure the maximum difference in protein expression between samples. In the Label-free proteome analysis, about 3,331 proteins were unequivocally identified in liver samples (Figures 4A–C). The first two components of the PCA explained 34.2% of the total variance (20.1 and 14.1% for PC1and PC2, respectively), and this analysis clearly separated the three groups along the PC1 (Figure 5A). According to the abundance of protein in three groups (Figure 4D), the proteins with fold change more than 1.5 and *P*-value below 0.05 (using the Benjamini-Hochberg multiple testing correction test) were considered to be significantly differentially expressed proteins (DEPs). We found that 59 of DEPs were upregulated and 93 downregulated in the HD compared to CD, whereas 52 were upregulated and 51 downregulated in B3D compared to the HD (Figures 4E–G, 5B). As proteases with similar expression patterns are usually functionally related, we used the heatmap with Euclidean distance to perform hierarchical clustering of the DEPs and samples simultaneously, according to the DEPs in HD group and/or B3D group.

Cluster 1 consisted of 47 DEPs, both in the HD group and the B3D group (Figure 5C). Notably, aconitate hydratase_A0A6I9PAY1 (involved in the pathway: tricarboxylic acid cycle, glyoxylate, and dicarboxylate metabolism) and gamma-enolase-like_A0A6I9PPR6 (glycolysis, gluconeogenesis, and methane metabolism) were downregulated in the HD group and upregulated in the B3D group. Also, flotillin_A0A484D865 (insulin signaling pathway) and protein phosphatase 1 regulatory subunit 1B_A0A4Z2J752 (cAMP signaling pathway) were upregulated in the HD group and downregulated in the B3D group.

Cluster 2 contained 13 of the upregulated and 16 of the downregulated DEPs in the HD group (Figure 5D). Notably, lysosomal alpha-glucosidase-like_A0A6I9NDC6 (galactos, starch and sucrose metabolism), GDH/6PGL endoplasmic bifunctional protein_A0A6I9P1U9 (pentose phosphate pathway), 3-hydroxy-3-methylglutaryl coenzyme A synthase_A0A6J2QQI0 (butanoate metabolism and PPAR signaling pathway) and apolipoprotein Ea_G3NCX8 (cholesterol metabolism) were upregulated. ras-related GTP-binding protein_A0A6I9MV42 (mTOR signaling pathway) and alkylglycerone-phosphate synthase_A0A6J2S0A6 (ether lipid metabolism) were downregulated.

Cluster 3 contained 32 of the upregulated DEPs and 25 of the downregulated DEPs in the B3D group (Figure 5E). Notably, NTP_transferase domain-containing protein_A0A6A5E1E9 (fructose and mannose metabolism), aldehyde dehydrogenase family 16 member A1_A0A6J2Q5H6 (fatty acid degradation and glycerolipid metabolism), apolipoprotein E_F5BZM3 (cholesterol metabolism) and glucose-6-phosphate isomerase_G1FKE6 (glycolysis/gluconeogenesis) were upregulated. The FMN hydroxy acid dehydrogenase domain-containing protein_A0A484CSB3 (glyoxylate and dicarboxylate metabolism), NADH-ubiquinone oxidoreductase subunit_A0A484DPP8 (oxidative phosphorylation), perilipin_A0A6A5DXU6 and perilipin_A0A6J2RM79 (both PPAR signaling pathway), acyl-coenzyme A thioesterase 1-like_A0A6I9N8B3 (fatty acid elongation and biosynthesis of unsaturated fatty acids), and Acetyl-CoA carboxylase_A0A6J2R3I9 (fatty acid biosynthesis) were downregulated.

By summarizing these pathways, we found that the most strongly influenced pathways between the CD, HD, and B3D groups were fatty acid biosynthesis, insulin signaling pathway, and AMPK signaling pathway (Figures 4H,I, 5F).

### Dietary BAs Improved the Lipid Metabolism by Decreasing Lipogenesis and Increasing Lipolysis in the Liver

**Figure 6:**
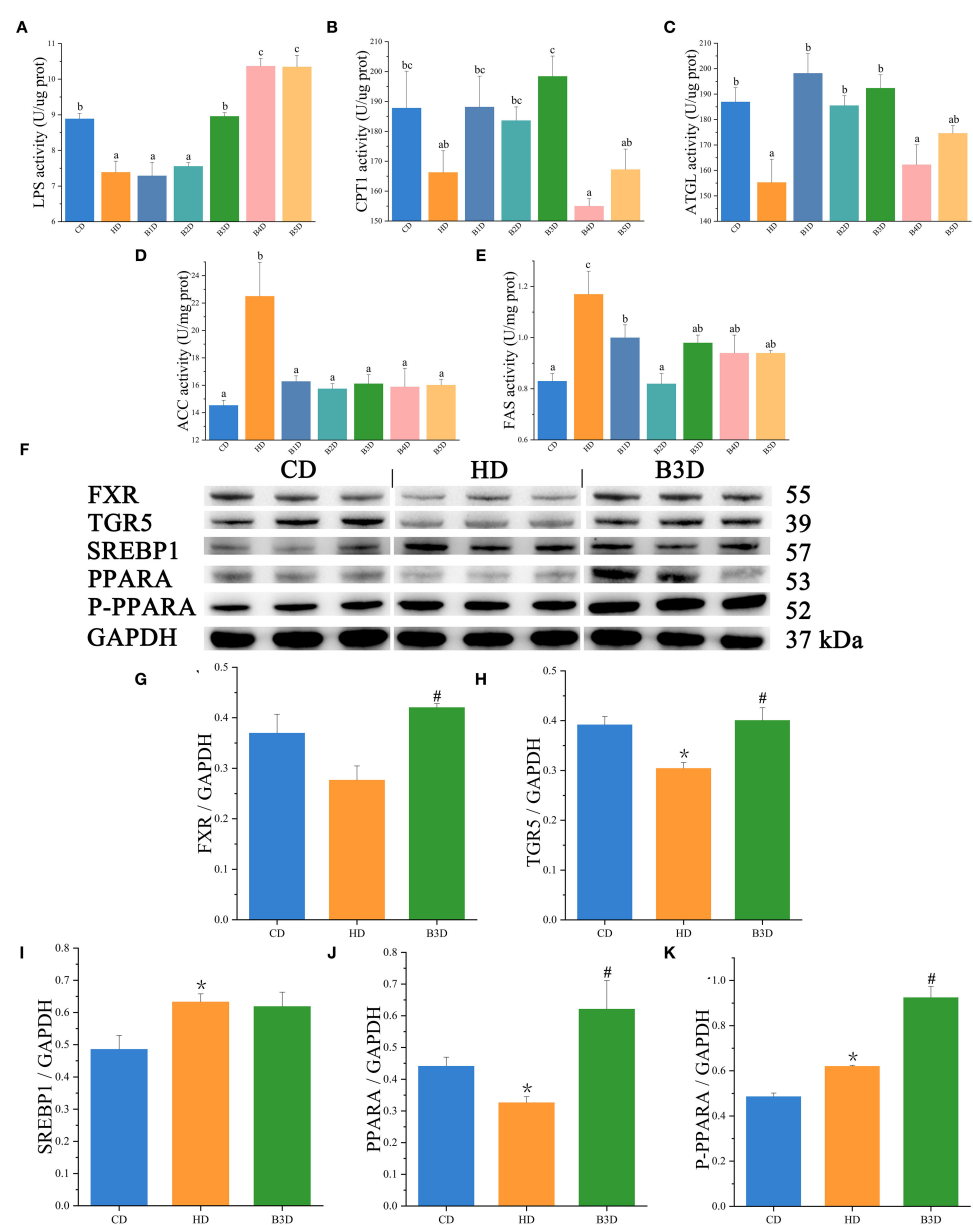
The activities of enzymes and expression of proteins in the liver samples. **(A–E)** the activities of LPS (lipase), CPT1 (carnitine palmitoyltransferase 1), ATGL (triglyceride lipase), ACC (acetyl-CoA carboxylase), and FAS (fatty acid synthase) in fish (*n* = 6). **(F)** the Western Blot analysis of SREBP1 (sterol responsive element binding protein 1), PPARA (peroxisome proliferator-activated receptor alpha), P-PPARA, and GAPDH in the liver (*n* = 3). **(G–K)** the relative quantification of FXR, TGR5, SREBP1, PPARA, and P-PPARA proteins normalized to the GAPDH level (*n* = 3). Values are presented as means with SD, where significant (*p* < 0.05) differences between groups are indicated by different letters. “*” indicates significant (*p* < 0.05) differences in the HD group (compared to the CD group), and “#” indicates significant (*p* < 0.05) differences in the B3D group (compared to the HD group).

To further explore the lipid metabolism in response to different diets, we measured the activities of enzymes, expression of genes, and proteins associated with lipid metabolism in liver samples. For ELISA assay, in the HD group, the activities of lipolysis enzymes (LPS and ATGL) were significantly decreased, whereas the activities of lipogenesis enzymes (ACC and FAS) were significantly increased (all *p* < 0.05, Figures 6A–E). After BAs intervention, the activities of lipolysis enzymes (LPS, CPT1, and ATGL) were increased in most BD groups, and the activities of lipogenesis enzymes (ACC and FAS) were decreased in all BD groups (Figures 6A–E). For Western Blot analyses, in the HD group, the expression of SREBP1 and P-PPARA proteins was significantly increased, while the expression of TGR5 and PPARA was significantly decreased (all *p* < 0.05, Figures 6F–K). In the B3D group, the expression of FXR, TGR5, PPARA and P-PPARA was significantly increased (Supplementary Table S2).

**Figure 7:**
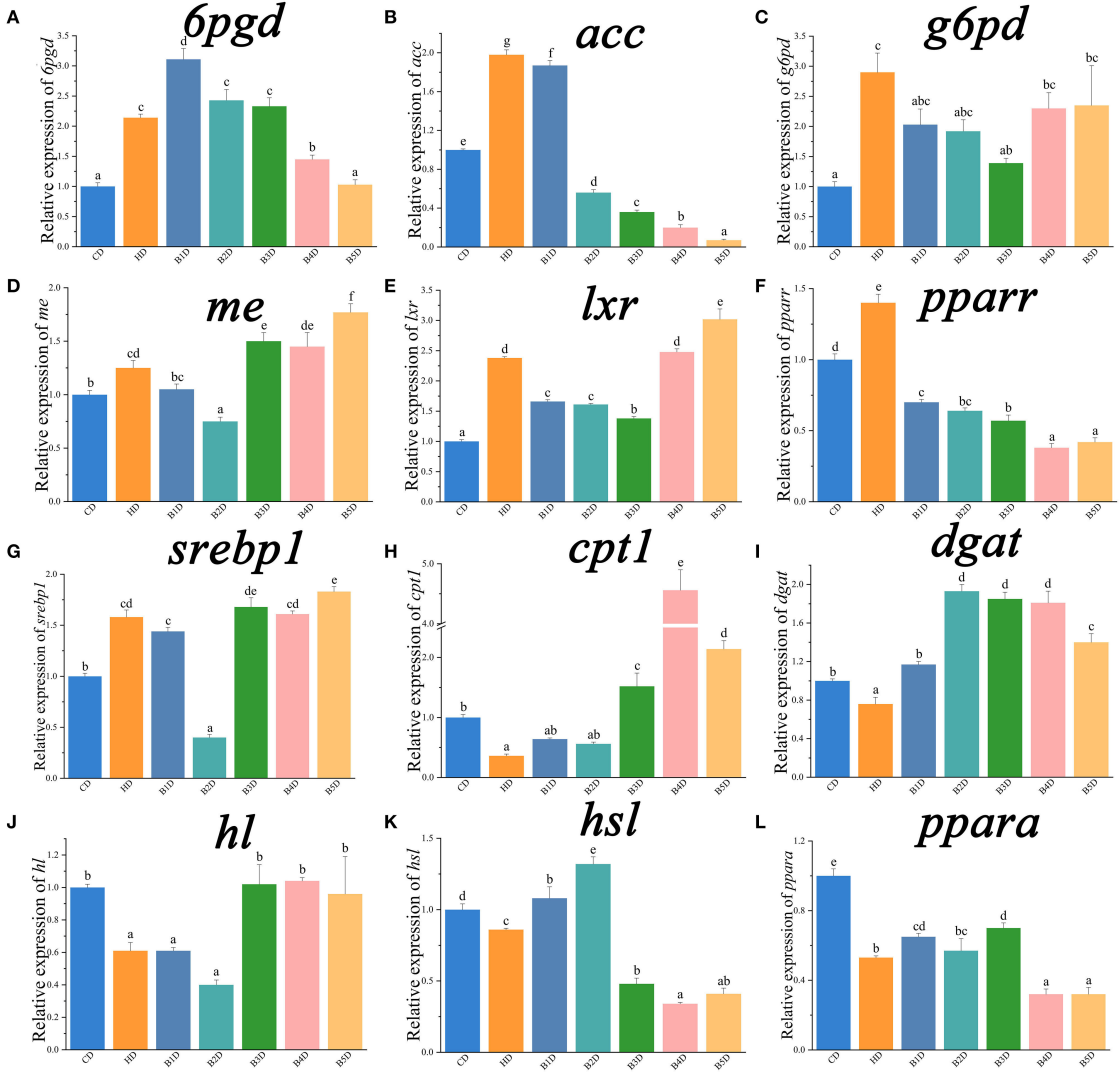
The relative expression of genes associated with lipid metabolism in the liver (part 1). *6pgd*, 6-phosphogluconate dehydrogenase; *acc*, acetyl-CoA carboxylase; *g6pd*, glucose 6-phosphate dehydrogenase; *me*, malic enzyme; *lxr*, liver X receptor alpha; *pparr*, peroxisome proliferator activated receptor gamma; *cpt1*, carnitine palmitoyltransferase 1; *dgat*, acyl CoA diacylglycerol acyltransferase 2; *hl*, hepatic lipase; *hsl*, hormone-sensitive lipase. Data are normalized to *18s* (18S ribosomal RNA) and β*-actin* as the reference genes and presented as a fold change in relation to the control group (CD, set as 1) (*n* = 6). Values are presented as means with SD, where significant (*p* < 0.05) differences between groups are indicated by different letters.

**Figure 8:**
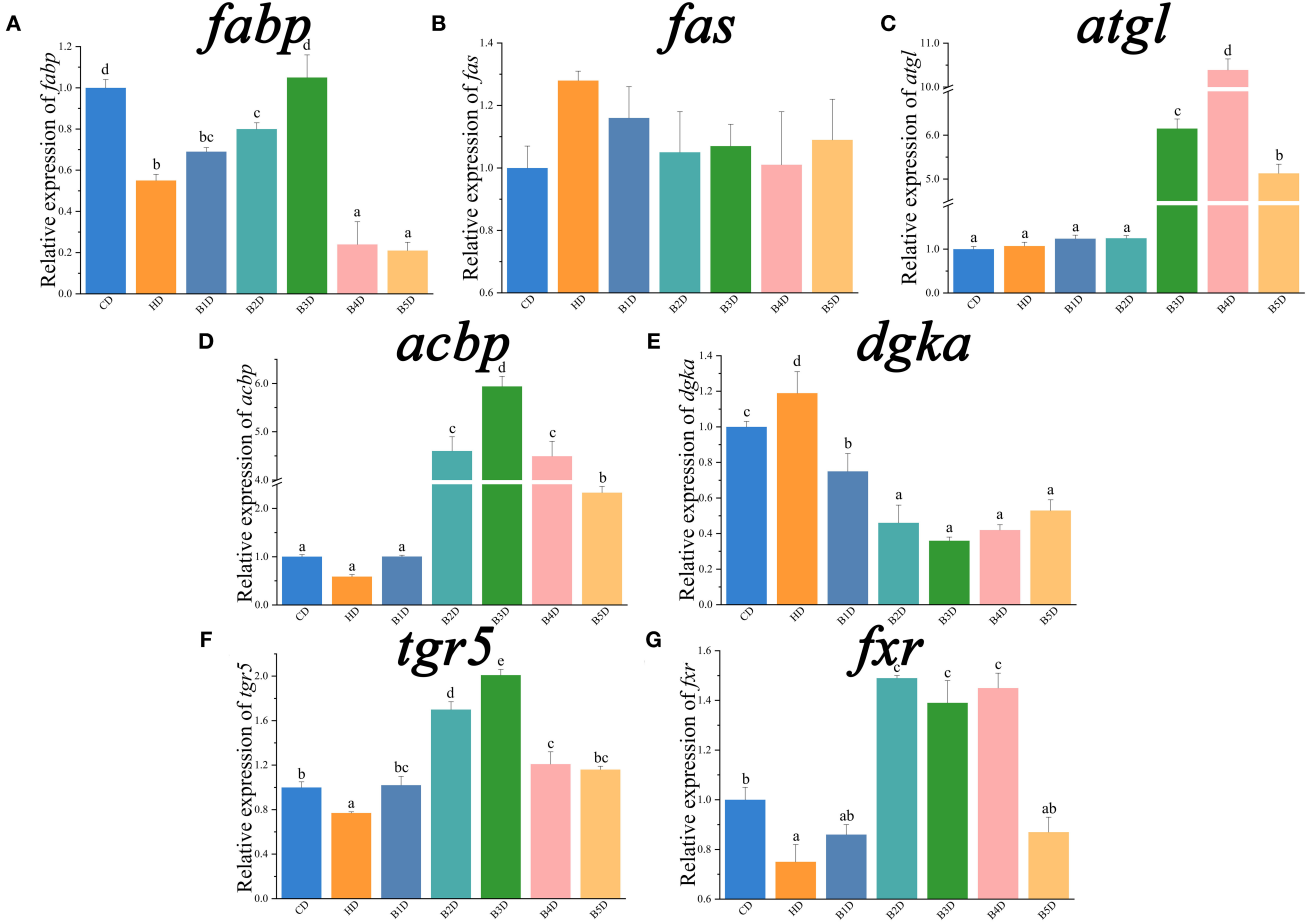
The relative expression of genes associated with lipid metabolism in the liver (part 2). *fabp*, fatty acid-binding protein; *fas*, fatty acid synthase; *atgl*, adipose triglyceride lipase; *acbp*, acyl-CoA binding protein; *dgka*, diacylglycerol kinase alpha; *tgr5*, G protein-coupled bile acid receptor 1; *fxr*, farnesoid X receptor. Data are normalized to *18s* and β*-actin* as the reference genes and presented as a fold change in relation to the CD (*n* = 6). Values are presented as means with SD, where significant (*p* < 0.05) differences between groups are indicated by different letters.

For qPCR analyses, the expression of *il1*β, *tnf*α, *cxcl8*, lipogenesis genes (*6pgd, acc, g6pd*, and *me*) and their transcriptional factors (*lxr, pparr*, and *srebp1*) was significantly increased in the HD group (Table 3; Figures 7A–G), whereas the expression of *il10*, lipolysis genes (*cpt1, dgat, hl*, and *hsl*) and their transcriptional factors (*ppara*), as well as fatty acid uptake genes (*fabp*), was significantly decreased (all *p* < 0.05, Figures 7H–L, 8A). After the BAs intervention, expression of *il1*β, *tnf*α, *cxcl8*, lipogenesis genes (*acc, fas, g6pd*) and their transcriptional factors (*pparr*) was significantly decreased, whereas the expression of *il10*, lipolysis (*atgl, cpt1*, and *dgat*) and fatty acid uptake (*acbp*) genes was significantly increased in most BD groups (all *p* < 0.05, Figures 7A–L, 8A–E). In addition, the expression of BAs receptors *tgr5* and *fxr* genes was significantly decreased in the HD group, and both increased in most BD groups (Figures 8F,G). From the perspective of genes, proteins and enzymes, these results overall indicated that HD impaired the lipid metabolism by increasing lipogenesis and decreasing lipolysis, and BD improved lipid metabolism by decreasing lipogenesis and increasing lipolysis.

**Table 3:** The relative expression of genes associated with inflammatory cytokines and chemokines in the liver.

**Relative expression of genes**							
	**CD**	**HD**	**B1D**	**B2D**	**B3D**	**B4D**	**B5D**
*tnfα*	1.00 ± 0.049^b^	1.86 ± 0.096^d^	1.48 ± 0.074^c^	1.76 ± 0.018^d^	0.63 ± 0.029^a^	0.44 ± 0.031^a^	1.87 ± 0.171^d^
*il1β*	1.01 ± 0.080^a^	2.05 ± 0.213^b^	1.77 ± 0.102^b^	1.79 ± 0.079^b^	1.25 ± 0.041^a^	1.71 ± 0.118^b^	1.74 ± 0.136^b^
*il10*	1.00 ± 0.065^d^	0.33 ± 0.008^a^	0.54 ± 0.047^b^	0.52 ± 0.026^b^	1.46 ± 0.032^e^	0.85 ± 0.044^c^	0.45 ± 0.039^ab^
*cxcl8*	1.00 ± 0.044^a^	2.89 ± 0.11^c^	2.34 ± 0.255^b^	1.34 ± 0.051^a^	1.19 ± 0.101^a^	2.57 ± 0.057^bc^	2.87 ± 0.175^c^

*tnfα, tumor necrosis factor-alpha; il1β, interleukin 1β; cxcl8, CXC chemokine ligand 8. Data are normalized to 18s (18S ribosomal RNA) and β-actin as the reference genes and presented as a fold change in relation to the control group (CD, set as 1) (n = 6). Values are presented as means with SD, where significant (p < 0.05) differences between groups are indicated by different letters*.

## Discussion

Firstly, we explored the effects of HD and BAs-supplemented diets on the growth performance of hybrid grouper. Following a previous study (4), the high-lipid diet model of hybrid grouper was established using a diet containing more than 15% fat in this study (HD). Although the FCR and FI were significantly decreased in the HD group, growth rate indicators (BW^F^, WGR, and SGR) were not affected in hybrid grouper. In this species, one study showed that fish fed a diet containing 10% lipid had the highest WGR, whereas 13% of dietary lipids significantly decreased it (23); another study showed that increasing the dietary lipid level from 7 to 14% did not significantly affect the growth rate indicators (24). In combination with our study, these results indicate that a large number of variables (such as diet composition, duration of the experiment, fish genotype, etc.) influence the lipid tolerance of hybrid grouper. This has important implications for the interpretation of data and comparison of these types of studies. In our study, after administering gradients of BAs under the HD conditions, hybrid grouper first exhibited an increased and then decreased growth rate. The addition of a suitable level of BAs also promoted the growth in other fish species, but the optimal level varied among different fish species: 900 or 475 mg kg^−1^ in largemouth bass (9, 10), 300 mg kg^−1^ in yellow croaker (11), 80 mg kg^−1^ in grass carp (12) and 150 mg kg^−1^ in tilapia (13). These studies also found that high levels of BAs supplementation had negative effects on the fish growth performance. Therefore, we conclude that hybrid grouper fed a high-fat diet responded very well to the 900 mg kg^−1^ BAs supplementation in terms of growth performance.

Following this, we explored the effects of HD on lipid accumulation in hybrid grouper. The HD caused a significant increase in lipid deposition, especially in the liver, which is in agreement with previous studies in hybrid grouper (4), tiger puffer (5), yellow catfish (6), and mice (7). In this study, supplementation of BAs reduced the accumulation of lipids in the liver of hybrid grouper, resulting in better hepatic health, decreased crude lipid levels, and improved biochemical parameters. Correspondingly, supplementation of exogenous BAs also successfully reverted the effects of HD in humans (17), yellow croaker (11) and largemouth bass (10). In agreement with our study, high levels of BAs supplementation increased lipid accumulation in the liver of yellow croaker and largemouth bass (9, 11). Our results showed that an HD diet supplemented with 900 mg kg^−1^ BAs produced the lowest hepatic lipid deposition.

We also explored the effects of HD and BD on the lipid metabolism of fish. In partial agreement with our results, the content of serum HDL was significantly decreased, while LDL was significantly increased, in response to a different hybrid grouper (*Epinephelus fuscoguttatus*♀ × *E. polyphekadion*♂) fed an HD (25). However, the BAs supplementation did not affect the content of HDL and LDL in the serum of tiger puffer (5) and largemouth bass (9), nor did it affect the TG and T-CHO content in the liver of largemouth bass (9). These discrepancies among studies could be caused by a number of different variables, such as doses (11, 15) and kinds (9) of BAs, as well as the basal dietary content of lipids, carbohydrates and proteins (5, 10, 26). Further trials are needed to clarify this. Overall, our results indicated that the HD diet impaired the lipid metabolism in the liver, and these adverse effects were apparently reversed with the addition of BAs.

To obtain more precise data, we used the proteome approach to explore the key pathways behind the BAs and lipid metabolism in the liver. The protein profiles differed among the CD, HD and B3D groups, which suggested shifts in the dominant function of organism protein after dietary lipids or BAs supplementation. Previous studies found that supplementation of BAs activated the AMPK pathway in largemouth bass on a high starch diet (10), and improved the insulin sensitivity of mice on an HD diet (17). In line with these observations, in this study we found that among many key functional pathways that were changed, the most strongly influenced pathways were fatty acid biosynthesis, insulin signaling pathway, and the AMPK signaling pathway between three groups.

Moreover, we explored how the BAs supplementation regulated the lipid metabolism. SREBP1 is a transcription factor that regulates multiple genes involved in fatty acid and lipid synthesis, including *acc* and *fas* (21). The HD group exhibited increased expression of lipogenesis genes (*6pgd, acc, g6pd*, and *me*) and proteins (SREBP1), and activities of lipogenesis enzymes (ACC and FAS) compared to the CD group, while most of the BD groups exhibited an opposite trend. As BAs negatively regulate the gene expression of *srebp1* in both fish and mammals (5, 16, 27), our results showed that supplementation of BAs inhibited the lipogenesis induced by HD. This further confirmed the indications of the proteome analysis, which suggested that lipogenesis was strongly affected. On the other hand, suppression of SREBP1 induces the expression of PPARA and its target genes to promote free fatty acid oxidation and lipolysis (28). In agreement with the downregulated/upregulated mRNA expression levels of *ppara* in large yellow croaker fed high-fat/BAs treatment diets, respectively (11), our study showed that hybrid grouper fed an HD also exhibited reduced expression of lipolysis genes (*cpt1, dgat, hl*, and *hsl*) and activities of lipolysis enzymes (LPS and ATGL), while these pathways were enhanced in most BD groups. Comparable to other studies in fish (5, 10–12), we also found that dietary BAs inclusion improved the hepatic lipid metabolism significantly by enhancing the hepatic lipolysis, inhibiting lipogenesis, and regulating associated transcriptional factors.

In addition, BAs are known to regulate lipid and glucose homeostasis through activation of the FXR and TGR5 signaling pathways (29). High hepatic expression of FXR protected against hepatic steatosis and elevated TG through the induction of lipolytic target genes in mice (27, 30), while TGR5 activated PPARA to increase mitochondrial oxidative phosphorylation and energy metabolism, as well as reduce obesity in humans (31). In the present study, the low expression of *fxr* gene, *tgr5* gene and protein in the HD group, and high expression of FXR and TGR5 genes and proteins in the B3D group, might indicate that an HD diet impaired, but exogenous BAs activated, the FXR and TGR5 pathways, which altered the hepatic lipid metabolism. Notably, activation of FXR and TGR5 inhibited the level of pro-inflammatory cytokines and chemokines genes in this study and previous researches (16, 32). As a decrease of these inflammatory markers is associated with the improvement of fatty liver disease in mice and humans (33), these findings suggested that activation of FXR and TGR5 signaling might be the key step toward the BAs-induced lipid-lowering outcome (in hybrid grouper). Further studies are needed to elucidate this mechanism.

## Conclusion

In summary, the present study showed that high dietary lipids induced lipid accumulation and impaired lipid metabolism in the liver of hybrid grouper. Supplementation of BAs promoted growth performance and reduced lipid accumulation in fish. In addition, BAs treatment improved hepatic lipid metabolism by enhancing hepatic lipolysis, inhibiting lipogenesis, and regulating associated transcriptional factors. Meanwhile, the regulatory effects of dietary BAs on lipid metabolism might be achieved through the FXR and TGR5 signaling pathways. The optimal supplementation level of BAs to a high-fat diet is 900 mg kg^−1^ in hybrid grouper. The findings of present study would help to develop the new feed additives to improve lipid deposition in fish. In addition, these data may also contribute to the understanding of the specific mechanism via which exogenous BAs improve the lipid metabolism in animals. In the future, evaluation of side effects of supplementation of BAs and optimal dosage for animals will require more trials before it can become a routine addition. However, preliminary studies do show a promising efficacy of TCA in the treatment of obesity, and other metabolic disorders such as fatty liver disease.

## Data Availability Statement

The datasets presented in this study can be found in online repositories. The names of the repository/repositories and accession number(s) can be found in the article/Supplementary Material.

## Ethics Statement

The protocol was approved by the Animal Ethical and Welfare Committee of Guangdong Ocean University (Guangdong, China), processing ID: GDOU-AEWC- 20180063.

## Author Contributions

JX: methodology, validation, formal analysis, investigation, data curation, and writing-original draft. XL: investigation and data curation. XY: investigation and writing-original draft. SX: conceptualization, resources, writing-review and editing, and visualization. SC: conceptualization, investigation, and writing-review and editing. SZ: conceptualization and writing-review and editing. JC: conceptualization, project administration, and funding acquisition. BT: conceptualization, writing-review and editing, project administration, and funding acquisition. All authors read and approved the final manuscript.

## Funding

This work was supported by the National Key R&D Program of China (2019YFD0900200), the National Natural Science Foundation of China (No. 31772864), the China Agriculture Research System of MOF and MARA (CARS-47), Science and Technology Project of Zhanjiang (2020A05003), and the Natural Science Foundation of Guangdong Province (2018A030313154 and 2020A1515011129).

## Conflict of Interest

The authors declare that the research was conducted in the absence of any commercial or financial relationships that could be construed as a potential conflict of interest.

## Publisher's Note

All claims expressed in this article are solely those of the authors and do not necessarily represent those of their affiliated organizations, or those of the publisher, the editors and the reviewers. Any product that may be evaluated in this article, or claim that may be made by its manufacturer, is not guaranteed or endorsed by the publisher.

## Abbreviations

*6pgd*: 6-phosphogluconate dehydrogenase
*acbp*: acyl-CoA binding protein
ACC: acetyl-CoA carboxylase
ATGL: triglyceride lipase
BAs: bile acids
BD: BAs diets
CD: control diet
CPT1: carnitine palmitoyltransferase 1
*dgat*: acyl CoA diacylglycerol acyltransferase 2
*dgka*: diacylglycerol kinase alpha
*fabp*: fatty acid-binding protein
FAS: fatty acid synthase
FCR: feed conversion ratio
FI: feed intake
FXR: farnesoid X receptor
*g6pd*: 6-phosphate dehydrogenase
HD: high lipid diet
HDL: high-density lipoprotein cholesterol
*hl*: hepatic lipase
HSI: hepatosomatic index
*hsl*: hormone-sensitive lipase
IOD: integrated optical density
LDL: low-density lipoprotein cholesterol
LPS: lipase
*lxr*: liver X receptor alpha
*me*: malic enzyme
NAFLD: non-alcoholic fatty liver disease
*ppara*: peroxisome proliferator-activated receptor alpha
*pparr*: peroxisome proliferator-activated receptor gamma
SGR: specific growth rate
SHP: small heterodimer partner
SREBP1: sterol responsive element binding protein 1
TG, triglycerides, TGR5: G protein-coupled bile acid receptor 1
T-CHO: total cholesterol
VSI: viscerasomatic index
WGR: weight gain rate.

## References

[1] Yin B Liu H Tan B Dong X Chi S Yang Q. MHC II-PI3K/Akt/mTOR signaling pathway regulates intestinal immune response induced by soy glycinin in hybrid grouper: protective effects of sodium butyrate. Front Immunol. (2020) 11:615980. 10.3389/fimmu.2020.615980 33537033 PMC7849651

[2] National Research Council. Nutrient Requirements of Fish and Shrimp. Washington: National Academies Press (2011).

[3] Wang J Han SL Li LY Lu DL Limbu SM Li DL. Lipophagy is essential for lipid metabolism in fish. Sci Bull. (2018) 63:879–82. 10.1016/j.scib.2018.05.026 36658967

[4] Zou C Su N Wu J Xu M Sun Z Liu Q. Dietary Radix Bupleuri extracts improves hepatic lipid accumulation and immune response of hybrid grouper (Epinephelus lanceolatusmale symbol x Epinephelus fuscoguttatusfemale symbol). Fish Shellfish Immunol. (2019) 88:496–507. 10.1016/j.fsi.2019.02.052 30826414

[5] Liao ZB Sun B Zhang QG Jia LL Wei YL Liang MQ. Dietary bile acids regulate the hepatic lipid homeostasis in tiger puffer fed normal or high-lipid diets. Aquaculture. (2020) 519:734935. 10.1016/j.aquaculture.2020.734935

[6] Zhang DG Zhao T Hogstrand C Ye HM Xu XJ Luo Z. Oxidized fish oils increased lipid deposition via oxidative stress-mediated mitochondrial dysfunction and the CREB1-Bcl2-Beclin1 pathway in the liver tissues and hepatocytes of yellow catfish. Food Chem. (2021) 360:129814. 10.1016/j.foodchem.2021.129814 34023714

[7] Zheng X Huang F Zhao A Lei S Zhang Y Xie G. Bile acid is a significant host factor shaping the gut microbiome of diet-induced obese mice. BMC Biol. (2017) 15:120. 10.1186/s12915-017-0462-7 29241453 PMC5731064

[8] Sayin SI Wahlstrom A Felin J Jantti S Marschall HU Bamberg K. Gut microbiota regulates bile acid metabolism by reducing the levels of tauro-beta-muricholic acid, a naturally occurring FXR antagonist. Cell Metab. (2013) 17:225–35. 10.1016/j.cmet.2013.01.003 23395169

[9] Yin P Xie SW Zhuang ZX He XS Tang XP Tian LX. Dietary supplementation of bile acid attenuate adverse effects of high-fat diet on growth performance, antioxidant ability, lipid accumulation and intestinal health in juvenile largemouth bass (*Micropterus salmoides*). Aquaculture. (2021) 531:735864. 10.1016/j.aquaculture.2020.735864

[10] Yu H Zhang L Chen P Liang X Cao A Han J. Dietary bile acids enhance growth, and alleviate hepatic fibrosis induced by a high starch diet via AKT/FOXO1 and cAMP/AMPK/SREBP1 pathway in *Micropterus salmoides*. Front Physiol. (2019) 10:1430. 10.3389/fphys.2019.01430 31824338 PMC6882294

[11] Ding T Xu N Liu YT Du JL Xiang XJ Xu D. Effect of dietary bile acid (BA) on the growth performance, body composition, antioxidant responses and expression of lipid metabolism-related genes of juvenile large yellow croaker (*Larimichthys crocea*) fed high-lipid diets. Aquaculture. (2020) 518:734768. 10.1016/j.aquaculture.2019.734768

[12] Zhou JS Chen HJ Ji H Shi XC Li XX Chen LQ. Effect of dietary bile acids on growth, body composition, lipid metabolism and microbiota in grass carp (*Ctenopharyngodon idella*). Aquac Nutr. (2018) 24:802–13. 10.1111/anu.12609

[13] Jiang M Wen H Gou GW Liu TL Lu X Deng DF. Preliminary study to evaluate the effects of dietary bile acids on growth performance and lipid metabolism of juvenile genetically improved farmed tilapia (*Oreochromis niloticus*) fed plant ingredient-based diets. Aquac Nutr. (2018) 24:1175–83. 10.1111/anu.12656

[14] Gu M Bai N Kortner TM. Taurocholate supplementation attenuates the changes in growth performance, feed utilization, lipid digestion, liver abnormality and sterol metabolism in turbot (Scophthalmus maximus) fed high level of plant protein. Aquaculture. (2017) 468:597–604. 10.1016/j.aquaculture.2016.11.022

[15] Romano N Kumar V Yang G Kajbaf K Rubio MB Overturf K. Bile acid metabolism in fish: disturbances caused by fishmeal alternatives and some mitigating effects from dietary bile inclusions. Rev Aquac. (2020) 12:1792–817. 10.1111/raq.12410

[16] Chavez-Talavera O Tailleux A Lefebvre P Staels B. Bile acid control of metabolism and inflammation in obesity, type 2 diabetes, dyslipidemia, and nonalcoholic fatty liver disease. Gastroenterology. (2017) 152:1679–94 e1673. 10.1053/j.gastro.2017.01.055 28214524

[17] Schaap FG Trauner M Jansen PL. Bile acid receptors as targets for drug development. Nat Rev Gastroenterol Hepatol. (2014) 11:55–67. 10.1038/nrgastro.2013.151 23982684

[18] Thomas C Pellicciari R Pruzanski M Auwerx J Schoonjans K. Targeting bile-acid signalling for metabolic diseases. Nat Rev Drug Discov. (2008) 7:678–93. 10.1038/nrd2619 18670431

[19] Donepudi AC Boehme S Li F Chiang JY. G-protein-coupled bile acid receptor plays a key role in bile acid metabolism and fasting-induced hepatic steatosis in mice. Hepatology. (2017) 65:813–27. 10.1002/hep.28707 27351453 PMC5195921

[20] Xu H Zhang Q Kim SK Liao Z Wei Y Sun B. Dietary taurine stimulates the hepatic biosynthesis of both bile acids and cholesterol in the marine teleost, tiger puffer (Takifugu rubripes). Br J Nutr. (2020) 123:1345–56. 10.1017/S0007114520000161 31959268

[21] Xu J Xie S Chi S Zhang S Cao J Tan B. Short-term dietary antibiotics altered the intestinal microbiota and improved the lipid metabolism in hybrid grouper fed medium and high-lipid diets. Aquaculture. (2021) 547:737453. 10.1016/j.aquaculture.2021.737453

[22] Ma J Chen T Wu S Yang C Bai M Shu K. iProX: an integrated proteome resource. Nucleic Acids Res. (2019) 47:D1211–7. 10.1093/nar/gky869 30252093 PMC6323926

[23] Li S Li Z Chen N Jin P Zhang J. Dietary lipid and carbohydrate interactions: implications on growth performance, feed utilization and non-specific immunity in hybrid grouper (*Epinephelus fuscoguttatus* ♀ × *E. lanceolatus* ♂). Aquaculture. (2019) 498:568–77. 10.1016/j.aquaculture.2019.734351

[24] Rahimnejad S Bang IC Park J-Y Sade A Choi J Lee S-M. Effects of dietary protein and lipid levels on growth performance, feed utilization and body composition of juvenile hybrid grouper, *Epinephelus fuscoguttatus* × *E. lanceolatus*. Aquaculture. (2015) 446:283–9. 10.1016/j.aquaculture.2015.05.019

[25] Xie R-T Amenyogbe E Chen G Huang J-S. Effects of feed fat level on growth performance, body composition and serum biochemical indices of hybrid grouper (*Epinephelus fuscoguttatus* × *Epinephelus polyphekadion*). Aquaculture. (2021) 530:735813. 10.1016/j.aquaculture.2020.735813

[26] Peng XR Feng L Jiang WD Wu P Liu Y Jiang J. Supplementation exogenous bile acid improved growth and intestinal immune function associated with NF-kappaB and TOR signalling pathways in on-growing grass carp (*Ctenopharyngodon idella*): enhancement the effect of protein-sparing by dietary lipid. Fish Shellfish Immunol. (2019) 92:552–69. 10.1016/j.fsi.2019.06.047 31252043

[27] Schmitt J Kong B Stieger B Tschopp O Schultze SM Rau M. Protective effects of farnesoid X receptor (FXR) on hepatic lipid accumulation are mediated by hepatic FXR and independent of intestinal FGF15 signal. Liver Int. (2015) 35:1133–44. 10.1111/liv.12456 25156247 PMC4146754

[28] Watanabe M Houten SM Wang L Moschetta A Mangelsdorf DJ Heyman RA. Bile acids lower triglyceride levels via a pathway involving FXR, SHP, and SREBP-1c. J Clin Invest. (2004) 113:1408–18. 10.1172/JCI21025 15146238 PMC406532

[29] Jia W Xie G Jia W. Bile acid-microbiota crosstalk in gastrointestinal inflammation and carcinogenesis. Nat Rev Gastroenterol Hepatol. (2018) 15:111–28. 10.1038/nrgastro.2017.119 29018272 PMC5899973

[30] Mandimika T Paturi G De Guzman CE Butts CA Nones K Monro JA. Effects of dietary broccoli fibre and corn oil on serum lipids, faecal bile acid excretion and hepatic gene expression in rats. Food Chem. (2012) 131:1272–8. 10.1016/j.foodchem.2011.09.117

[31] Chiang JYL Ferrell JM. Bile acid receptors FXR and TGR5 signaling in fatty liver diseases and therapy. Am J Physiol Gastrointest Liver Physiol. (2020) 318:G554–73. 10.1152/ajpgi.00223.2019 31984784 PMC7099488

[32] Tian J-J Jin Y-Q Yu E-M Sun J-H Xia Y Zhang K. Farnesoid X receptor is an effective target for modulating lipid accumulation in grass carp, *Ctenopharyngodon idella*. Aquaculture. (2021) 534:736248. 10.1016/j.aquaculture.2020.736248

[33] Molinaro A Wahlstrom A Marschall HU. Role of bile acids in metabolic control. Trends Endocrinol Metab. (2018) 29:31–41. 10.1016/j.tem.2017.11.002 29195686

