# Supplementary material for: Protective Effects of Bile Acids Against Hepatic Lipid Accumulation in Hybrid Grouper Fed a High-Lipid Diet
Source: Front Nutr. 2022 Jan 25;9:813249. doi: 10.3389/fnut.2022.813249 (PMC8821168; doi:10.3389/fnut.2022.813249)
Supplement: Supplementary file 1 [file Table_1.docx]

**SUPPLEMENTARY MATERIAL**

**2.1 Animals and diet preparation**

The hybrid grouper (*Epinephelus fuscoguttatus*♀ × *E. lanceolatus*♂) juveniles (before sex differentiation) were purchased from a local fish farm (Zhanjiang, China) and acclimated for 7 days by feeding with a commercial diet (50% crude protein, 10% crude lipid). Feeding was performed every day at 8:00 and 17:00 until the apparent satiation (about 3.0–5.0% of the fish body weight). During the experiment, the water temperature fluctuated from 28 to 30°C, the dissolved oxygen concentration was kept at > 7 mg/L, and ammonia and nitrate were kept at < 0.03 mg/L. 70% of the water in tanks was replaced daily. All feed ingredients were crushed into powder through a 380 mm mesh and combined. Fish oil, soy lecithin, and water were then added and mixed thoroughly. After pelletization and air drying for 2–3 days at room temperature, all prepared feed was kept in a refrigerator (-20°C) until use.

**2.2 Feeding experiment and sample collection**

At the end of the rearing period of 8 weeks, fish from each tank were anaesthetized with MS-222 (Sigma Aldrich, USA) at a concentration of 100 mg L^−1^. The number, final total weight of all fish in each tank, in addition to the final body weight (BW^F^) and length of three randomly selected fish, were recorded. After euthanizing the fish by trauma to the head and dissection, 12 specimens from each tank were selected randomly to obtain samples of the whole body (2 fish), blood (3 fish), muscle (2 fish) and liver (5 fish).

Muscle and whole-body samples from each tank were stored at -20°C for the composition analyses. Blood was obtained from the caudal vein using sterile syringes and then centrifuged (3000×g) at 4°C for 10 min to obtain the serum, which was quickly frozen in liquid nitrogen and stored at -80°C. The serum samples were used for measuring enzyme activity and biochemical indicators. Each liver was divided into seven parts: one was placed in 4% paraformaldehyde solution for histological examination, one was stored at -20°C for body composition analysis, and the remainder was quickly frozen in liquid nitrogen and stored at -80°C. The latter five liver samples were used for measuring the enzyme activity, testing biochemical indicators, determining gene and protein expression, and performing proteomic analysis.

# 2.3 Growth performance analyses

# The following growth parameters were calculated:

Survival rate (SR, %) = ﬁnal ﬁsh number / initial ﬁsh number × 100

Weight gain rate (WGR, %) = (final body weight - initial body weight) × 100 / initial body weight.

Specific growth rate (SGR, %) = 100 × (Ln final body weight - Ln initial body weight) / experiment duration in days.

Feed conversion ratio (FCR) = dry diet fed / wet weight gain.

Feed intake (FI, %) = 100 × diet fed / (final body weight + initial body weight) / (2 × the experimental duration in days).

Viscerasomatic index (VSI, %) = 100 × viscer wet weight / body wet weight.

Hepatosomatic index (HSI, %) = 100 × liver wet weight / body wet weight.

Condition factor (CF, g/cm^3^) = 100 × body weight / (body length^3^).

**2.7 The qPCR analyses**

The total RNA of the liver tissue was extracted using 1 ml of Trizol (TRI Reagent solution, Invitrogen, Carlsbad, CA, USA) according to the instructions previously described (Yin et al., 2020). The RNA quality and quantity were evaluated by electrophoresis in a 1.5 % agarose gel and NanoDrop 2000 spectrometer (Thermo Scientific, Delaware, USA). The cDNA sample of extracted total RNA was synthesized using the methods of PrimeScript^®^RT reagent Kit with gDNA Eraser (TaKaRa, Dalian, China) according to the manufacturer’s instructions. The amplicon lengths and reaction efficiency values for qPCR are shown in Table 1. The expression levels of genes were analyzed by LightCycler^®^480 II (Roche Diagnostics GmbH, USA) using 384-well plates. The qPCR reaction mixture contained the SYBR Premix Ex Taq™ (5 μL), ddH_2_O (3.6 μL), forward and reverse primers (0.4 μL, respectively), and cDNA template (1 μL). The qPCR protocol was: 5 min at 95°C; 40 cycles of 10 s at 95°C, 10 s at 58°C, and 10 s at 72°C; 5 min at 72°C. Each sample was studied in triplicate. Eight housekeeping genes (*β-actin, elongation factor 1α, glyceraldehyde 3-phosphate dehydrogenase, hypoxanthine* *phosphoribosyltransferase 1, ribosomal protein L8, 18S ribosomal RNA, β-2-microglobulin*, and *tubulin α*) were selected to test their transcriptional stability (Table S1). The geNorm (Center for Medical Genetics, Ghent, Belgium; https://genorm.cmgg.be/) was used to choose the best two internal reference genes, and then the geometric mean of expression levels of these two internal reference genes was used to calculate the relative expression of genes using the 2^-ΔΔCt^.

**2.9 Determining the levels of TCA in diets**

Samples of diets were weighed accurately and dipped in water for 4 h (vortexed for 1 min every 1 h). The solution was centrifuged at 13,200 rpm for 5 min. The supernatant was collected, 100 μl of it was mixed with 400 μl of methanol thoroughly, centrifuged again, and the resulting supernatant was subjected to the HPLC-MS/MS. The conditions for mass spectrometry detection, optimized to obtain the highest signal intensity, were as follows: mode=negative-ion mode; ion spray voltage=4500V; nebulizer gas pressure=55psi; curtain gas pressure=20psi; collision gas pressure=medium; turbo gas temperature=500°C; entrance potential=-10V; collision cell exit potential=-2V. Nitrogen gas was used as the collision gas in the multiple reaction monitoring mode. Regression equations between ‘Analyte Concentration’ (x; ng/ml) and ‘Analyte Peak Area’ (y; cps) for TCA was: y = 0.496 x + 0.378 (r = 0.9998). The calibration solution used for calibration of the system and quantitative results of TCA levels in diets are shown in Tables S2 and S3.

**2.10 The proteomic analyses**

***Protein extraction and digestion***

Proteins were extracted from liver samples of each experimental group (CD, HD, and B3D) as previously described (4). Three biological replicates were used. Briefly, 500 μL of Lysis Buffer (2% sodium deoxycholate, 50 mM ammonium bicarbonate, 75 mM sodium chloride) was added to samples, which were then ground. After placing samples on ice for 10 min at 2s on and 4s off at 15% power in a sonifier (Scientz-JY92, Ningbo Xinzhi biotechnology co., Ltd), the ground material was centrifuged at 10,000 g for 10 min at 4°C. By the method of acetone precipitation at -20℃, the precipitated proteins were air-dried and resuspended in Lysis Buffer, as previously described (2). The protein concentration was determined using a BCA assay. The resulting supernatants were incubated with DTT (final concentration to 10mM) at 56°C for 1 h to degrade the proteins, then alkylated with iodoacetamide (55 mM) at room temperature for 45 min in darkness. The protein solution (100 μg) was digested using trypsin (2.5 μg) at 37°C overnight. Peptides (digested protein) were desalted with a Strata X C18 column (Phenomenex, Torrance, CA, USA) and then vacuum-dried.

***Peptides fractionation***

The peptides were separated using a Shimadzu LC-20AB HPLC Pump system (Shimadzu, Kyoto, Japan) coupled with a high-pH RP column. The peptides were first reconstituted with 2 mL buffer A (5% ACN in water), and loaded onto a column containing 5 μm particles (Phenomenex, CA, USA). The peptides were separated with a gradient of 5% Buffer B (5% H_2_O in ACN) for 10 min, 5–35% Buffer B for 40 min, and 35–95% Buffer B for 1 min at a flow rate of 1 ml/min all the time. Then the system was maintained in 95% Buffer B for 3 min, followed by a decrease to 5% Buffer B within 1 min, and equilibration with 5% Buffer B for 10 min at a flow rate of 1 ml/min all the time. The elution process was monitored at 214 nm absorbance, and fractions were collected every 1 min. The eluted peptides were pooled as 20 fractions and concentrated by vacuum centrifugation.

***LC-MS/MS analyses***

Each fraction was resuspended in Buffer A (2% ACN and 0.1% formic acid, in water) and centrifuged at 20,000 × g for 10 min. The supernatant was loaded onto a C18 trap column on an LC-20 AD nano-HPLC instrument by the autosampler (Shimadzu, Kyoto, Japan). The peptides were eluted from the column and separated using an analytical C18 column (75 μm inner diameter) packed in-house. The 8%–35% Buffer B (2% water and 0.1% formic acid, in ACN) gradient was applied at 600 nl/min for 35 minutes, and 60% Buffer B for 5 min, 80% Buffer B for 5 min, and then 5% Buffer B for 0.1 min, followed by a 10 min equilibration.

The eluted peptides underwent nanoelectrospray ionisation before being analysed by MS/MS (Orbitrap Fusion Lumos mass spectrometer; Thermo Fisher Scientific, San Jose, CA, USA) coupled with the nanoHPLC system. A data-dependent mode in the scan range of 350–1,800 m/z was carried out for the MS analyses, and the survey scans were captured at a mass resolution of 120,000 at 400 m/z by the Orbitrap analyzer. In the linear ion trap, ten of the most intense precursor ions were selected for secondary MS analysis under the mode of high-energy collision dissociation. The dynamic exclusion parameter included an exclusion count of 2 and an exclusion time of 30s.

***Protein identification, quantification and annotation***

The raw data files were processed and quantified by Proteome Discoverer software v2.1 (Thermo Fisher Scientific, Massachusetts, USA) and searched against the Uniprot *Perciformes*.fasta (299081sequences, release 2020_04) using the SEQUEST algorithm. The search parameters used were as follows: fixed modifications, including carbamidomethylation of cysteine (+57.02146 Da); variable modification, including oxidation of methionine (+15.99492 Da). Mass tolerance of precursor ions was 20 ppm, and fragment ions were set to ± 0.5 Da. Trypsin was set as the enzyme, allowing for 2 missed cleavages. Searches used a reversed sequence decoy strategy to control peptide false discovery and identifications were validated by Percolator software. False discovery rate (FDR) was set to 0.01 for proteins and peptide-spectrum matches (PSMs). Background correction was checked for protein quantification and normalization. For quantification, proteins with at least one unique peptide were considered for further analysis. Proteins with a fold change > 1.5 and a p-value < 0.05 were considered to be significantly differentially expressed proteins (DEPs). The tool Blast2GO (blast2go.com) was used to classify the DEPs identified according to the current GO annotation (March 2018; geneontology.org) (1). Furthermore, DEPs were mapped onto KEGG pathways using the KEGG Mapper tool (http://www.genome.jp/kegg) (3). The MS proteomics data have been deposited to the ProteomeXchange Consortium (http://proteomecentral.proteomexchange.org) via the iProX partner repository with the dataset identifier PXD027928.

**References**

1. Consortium, G. O. Gene ontology consortium: going forward. *J Nucleic acids research.* (2015). *43*:D1, D1049-D1056.

2. Crowell, A. M., Wall, M. J., & Doucette, A. A. Maximizing recovery of water-soluble proteins through acetone precipitation. *Anal Chim Acta.* (2013). *796*, 48-54. <https://doi.org/10.1016/j.aca.2013.08.005>.

3. Du, J., Yuan, Z., Ma, Z., Song, J., Xie, X., & Chen, Y. KEGG-PATH: Kyoto encyclopedia of genes and genomes-based pathway analysis using a path analysis model. *Mol Biosyst.* (2014). *10*:9, 2441-2447. <https://doi.org/10.1039/c4mb00287c>.

4. Unwin, R. D., Griffiths, J. R., & Whetton, A. D. Simultaneous analysis of relative protein expression levels across multiple samples using iTRAQ isobaric tags with 2D nano LC–MS/MS. *J Nature protocols.* (2010). *5*:9, 1574.

**Table S1** Primers designed for qPCR

| Target | Sequences Forward | Sequences Reverse | Fragment length (bp) | Primer efficiency (%) |
| --- | --- | --- | --- | --- |
| *6pgd* | GACCACGATGAGATGGCACA | TGTACCGTCAGAGTCCCTGT | 111 | 96.47 |
| *acc* | ACTGGGGTGGTTGCTGTGG | CCTTAATAGCTTGGGCTGTTTTG | 148 | 100.34 |
| *fas* | CGGGTGTCTACATTGGGGTG | GAATAGCGTGGAAGGCGTTT | 213 | 99.77 |
| *g6pd* | GCTTCACATCCTTGTATCTGCTC | GCGTTCCTTTCATTCTCCG | 246 | 95.96 |
| *me* | GAAGTTGTTCTACCGCTTGCTG | AGAGTCCTCGTGGTCTCCTGA | 122 | 98.99 |
| *atgl* | ATTGAGCACCTTCCACCCA | CCGAATCCATCCCACATCTT | 213 | 99.41 |
| *cpt1* | CTTCATCCAGATCGCCCTACA | GCCCTCACGGAACAAACG | 97 | 91.22 |
| *dgat* | CATCTTCTGCTTTGGTGCTTTC | GCATTTCCCGTCCCGTTA | 207 | 99.17 |
| *dgka* | CATCTTCTGCTTTGGTGCTTTC | GCATTTCCCGTCCCGTTA | 225 | 92.95 |
| *hl* | GTGGTCGGTGGATGGTATGA | TGCCAATGGTGCGGGTT | 155 | 94.56 |
| *hsl* | CAGCCTGGAGCCCGTTAT | TTGGCGGTGATGTAGCG | 153 | 92.41 |
| *acbp* | GTTTGAGAAGATGGCAGTGGAC | CCTTTGCGAGTGTGATGTAGG | 224 | 97.24 |
| *fabp* | GTTGTCACCTCCAAGACTCCG | GACCATTTTGCCACCCTCC | 129 | 105.68 |
| *lxr* | CAGAAGCAATGCAACAAAAGG | TCAGTGAAGTGGGCGAACC | 116 | 92.44 |
| *ppara* | CATCGACAATGACGCCCTC | GCCGCTATCCCGTAAACAAC | 135 | 99.51 |
| *pparr* | GCGCCAGACACACACAATTT | CACTCGATGTTTAGCGCTGC | 119 | 101.34 |
| *srebp1* | TGTATCCAACTGTTGAGCACCTG | CTGTGGCAGTGTGGTCCTAG | 196 | 99.85 |
| *fxr* | AGGTGCTTGTGAGTGCCATC | TTCCTCTGCGCTGTACTGTT | 98 | 94.91 |
| *tgr5* | ATGCCATCACCATACCGCTG | CCAGGCGATGCCTAAGATGA | 90 | 99.39 |
| *il1β* | CCAGCGTTGAGGGCAGAA | ATCGTCTCCAGATGTAAGGTT | 103 | 92.45 |
| *cxcl8* | TTCGACGAGCTCAAGAGTGAG | TTCGACGAGCTCAAGAGTGAG | 134 | 101.63 |
| *tnfα* | GCTGCGGCTCGAAGACAAT | CAGACGGTGCGGATGGAGT | 219 | 96.35 |
| *il10* | CAGCAGAGTCATTGTCATCTCC | GAGTGGCAATGATCTCAGTCTC | 117 | 98.64 |
| *actin* | TACGAGCTGCCTGACGGACA | GGCTGTGATCTCCTTCTGC | 239 | 96.28 |
| *ef1a* | TCCCACAGAAGCCCATGGTT | CCGACGGCTACGGTCTGTCT | 94 | 94.62 |
| *gapdh* | CACGAAGGGCATTCTGGGATA | CATCAGGTCGCAGACACGGTT | 175 | 98.83 |
| *hprt1* | GCGTGCTCAAAGGGGGTTAC | TCATTGGGATGGAACGGTCA | 90 | 102.05 |
| *rpl8* | CGTCAGGAAACTACGCCACA | TTTCTTGGAGCCTGAGGGGA | 83 | 98.22 |
| *18s* | AGCAACTTTAGTATACGCTATTG | CCTGAGAAACGGCTACCACATC | 221 | 94.69 |
| *b2m* | GGACAGTATGGCAAAGACAAC | GCTTGGTCAGATGGAAGTG | 156 | 99.25 |
| *tuba* | TACGATATCTGCCGCAGGAAC | ACGAAGGGACGCAGTGATG | 102 | 102.53 |

*6pgd*: 6-phosphogluconate dehydrogenase; *acc*: acetyl-CoA carboxylase; *fas*: fatty acid synthase; *g6pd*: glucose 6-phosphate dehydrogenase; *me*: malic enzyme; *atgl*: adipose triglyceride lipase; *cpt1*: carnitine palmitoyltransferase 1; *dgat*: acyl CoA diacylglycerol acyltransferase 2; *dgka*: diacylglycerol kinase alpha; *hl*: hepatic lipase; *hsl*: hormone-sensitive lipase; *acbp*; acyl-CoA binding protein; *fabp*: fatty acid binding protein; *lxr*: liver X receptor alpha; *ppar*a: peroxisome proliferator activated receptor alpha; *pparr*: peroxisome proliferator activated receptor gamma; *srebp1*: sterol-regulator element-binding protein 1; *fxr*: farnesoid X receptor; *tgr5*: G protein-coupled bile acid receptor 1; *il1β*: interleukin 1β; *tnfα*: tumor necrosis factor-alpha; *actin*: β-actin; *ef1a*: elongation factor 1α; *gapdh*: glyceraldehyde 3-phosphate dehydrogenase; *hprt1*: hypoxanthine phosphoribosyltransferase 1; *rpl8*: ribosomal protein L8; *18s*: 18S ribosomal RNA; *b2m*: β-2-microglobulin; *tuba*: tubulin α.

**Table S2** Accuracy of calibration solution in analyses of TCA in diets

| Sample Name | Sample Type | Peak Area | Analyze Peak Area (counts) | Analyze Peak Height (cps) | Analyze Concentration (ug/mL) | Accuracy (%) |
| --- | --- | --- | --- | --- | --- | --- |
| S0 | Standard | 0.00E+00 | 0 | 1.00E+04 | N/A | / |
| S1 | Standard | 3.35E+04 | 2.5 | 2.38E+04 | 2.07 | 82.80 |
| S2 | Standard | 1.45E+05 | 10 | 2.63E+04 | 10.4 | 104.00 |
| S3 | Standard | 1.57E+05 | 13 | 2.46E+04 | 12.1 | 93.08 |
| S4 | Standard | 3.57E+05 | 26 | 2.50E+04 | 28.1 | 108.08 |
| S5 | Standard | 1.53E+06 | 130 | 2.37E+04 | 130 | 100.00 |
| HD | Unknown | 1.31E+05 | N/A | 2.15E+04 | 11.6 | / |
| B1D | Unknown | 3.90E+05 | N/A | 2.16E+04 | 35.6 | / |
| B2D | Unknown | 7.21E+05 | N/A | 2.21E+04 | 65 | / |
| B3D | Unknown | 1.06E+06 | N/A | 2.25E+04 | 94.2 | / |
| B4D | Unknown | 4.46E+05 | N/A | 2.22E+04 | 39.8 | / |
| B5D | Unknown | 7.40E+05 | N/A | 2.58E+04 | 57.1 | / |

**Table S3** Quantitative results of TCA in diets

| Sample Name | Analyze Concentration (ug/mL) | Sample weight  (mg) | Sample solution volume (ml) | Sample solution concentration (mg/ml) | Concentration in diets (mg/kg) |
| --- | --- | --- | --- | --- | --- |
| HD | 11.6 | 356.9 | 4 | 89.2 | 130 |
| B1D | 35.6 | 362.1 | 4 | 90.5 | 393.3 |
| B2D | 65 | 394 | 4 | 98.5 | 659.9 |
| B3D | 94.2 | 424.3 | 4 | 106.1 | 888.1 |
| B4D | 39.8 | 265.8 | 8 | 33.2 | 1197.9 |
| B5D | 57.1 | 304.1 | 8 | 38 | 1502.1 |
